# Alterations in Gut Microbiota of Gestational Diabetes Patients During the First Trimester of Pregnancy

**DOI:** 10.3389/fcimb.2020.00058

**Published:** 2020-02-27

**Authors:** Shujuan Ma, Yiping You, Lingting Huang, Sisi Long, Jiayue Zhang, Chuhao Guo, Na Zhang, Xinrui Wu, Yanni Xiao, Hongzhuan Tan

**Affiliations:** ^1^Department of Epidemiology and Health Statistics, Xiangya School of Public Health, Central South University, Changsha, China; ^2^Department of Obstetrics, Hunan Provincial Maternal and Child Health Hospital, Changsha, China

**Keywords:** biomarker, early prediction, gestational diabetes mellitus, gut microbiota, nested case-control study

## Abstract

**Background:** Dysbiosis of human gut microbiota is associated with a wide range of metabolic disorders, including gestational diabetes mellitus (GDM). Yet whether gut microbiota dysbiosis participates in the etiology of GDM remains largely unknown.

**Objectives:** Our study was initiated to determine whether the alternations in gut microbial composition during early pregnancy linked to the later development of GDM, and explore the feasibility of microbial biomarkers for the early prediction of GDM.

**Study design:** This nested case-control study was based upon an early pregnancy follow-up cohort (ChiCTR1900020652). Gut microbiota profiles of 98 subjects with GDM and 98 matched healthy controls during the early pregnancy (10–15 weeks) were assessed via 16S rRNA gene amplicon sequencing of V4 region. The data set was randomly split into a discovery set and a validation set, the former was used to analyze the differences between GDM cases and controls in gut microbial composition and functional annotation, and to establish an early identification model of GDM, then the performance of the model was verified by the external validation set.

**Results:** Bioinformatic analyses revealed changes to gut microbial composition with significant differences in relative abundance between the groups. Specifically, *Eisenbergiella, Tyzzerella 4*, and *Lachnospiraceae NK4A136* were enriched in the GDM group, whereas *Parabacteroides, Megasphaera, Eubacterium eligens* group, etc. remained dominant in the controls. Correlation analysis revealed that GDM-enriched genera *Eisenbergiella* and *Tyzzerella 4* were positively correlated with fasting blood glucose levels, while three control-enriched genera (*Parabacteroides, Parasutterella*, and *Ruminococcaceae UCG 002*) were the opposite. Further, GDM functional annotation modules revealed enrichment of modules for sphingolipid metabolism, starch and sucrose metabolism, etc., while lysine biosynthesis and nitrogen metabolism were reduced. Finally, five genera and two clinical indices were included in the linear discriminant analysis model for the prediction of GDM; the areas under receiver operating characteristic curves of the training and validation sets were 0.736 (95% confidence interval: 0.663–0.808) and 0.696 (0.575–0.818), respectively.

**Conclusions:** Gut bacterial dysbiosis in early pregnancy was found to be associated with the later development of GDM, and gut microbiota-targeted biomarkers might be utilized as potential predictors of GDM.

## Introduction

Gestational diabetes mellitus (GDM), defined as any degree of glucose intolerance initially diagnosed during pregnancy (ADA, 2019). It occurs ~5–20% of all pregnancies with rising prevalence (Zhu and Zhang, 2016), and is becoming a great threat to maternal and neonatal health, such as gestational hypertension, pre-eclampsia, cardiovascular disease and type 2 diabetes (T2DM) in the mothers (Metzger et al., 2008; Bellamy et al., 2009), as well as premature birth, fetal macrosomia, obesity, and T2DM in the offspring (Dabelea and Pettitt, 2001; Mitanchez, 2010). Considering the inordinate potential for harm from this illness, early detection and prevention are essential. However, no readily defined early diagnostic criterion or efficient prediction system is currently available for GDM (Kennelly and McAuliffe, 2016), and the explicit mechanism underlying its onset has not yet been fully clarified.

Microbes that reside in the human gut are increasingly recognized as one of the important contributors to host metabolism and health (Le Chatelier et al., 2013). Gut microbiota changes significantly during gestation (Koren et al., 2012) and may contribute to metabolic dysfunction during pregnancy, like GDM (Lv et al., 2018). Specific differences in gut microbiota between GDM patients and normoglycemic pregnant women were reported by several recent studies (Kuang et al., 2017; Mokkala et al., 2017; Cortez et al., 2018; Crusell et al., 2018; Wang et al., 2018a; Ye and Zhang, 2019). Increased abundance of *Klebsiella variicola* (Kuang et al., 2017), *Ruminococcus, Prevotella* (Cortez et al., 2018), *Desulfovibrio, Rothia* (Crusell et al., 2018), *Fusobacterium* (Wang et al., 2018a), *Blautia, Eubacterium hallii* group (Ye and Zhang, 2019), and reduced richness of *Bifidobacterium spp., Eubacterium spp*. (Kuang et al., 2017), *Bacteroides, Parabacteroides, Dialister, Akkermansia* (Cortez et al., 2018), *Marvinbryantia, Anaerosporobacter* (Crusell et al., 2018), and *Faecalibacterium* (Wang et al., 2018a; Ye and Zhang, 2019) in the gut were reported in GDM patients compared to normoglycemic controls. The only study that focused on changes in gut microbiota preceding diagnosis of GDM (15 of the total 75 overweight/obese women developed GDM) demonstrated that increased proliferation of the Ruminococcaceae family in the gut was associated with a higher potential of developing GDM (Mokkala et al., 2017). However, limited by cross-sectional study design and small sample size, the exact mechanisms involved in causing this significant shift in the dominant bacteria within the gut could not be clarified. In order to know more about the potential role of gut microbiota in the etiology of GDM, it is important to set the observation point before its onset, and transfer the study population focus from high-risk individuals to general pregnant women.

In the present nested case-control study, we evaluated women in the early stages of pregnancy from the general population in an effort to ascertain the bacteria involved during the gut flora transformation, which renders the subject susceptible to GDM. We also established an early identification model of GDM based on bacteria markers and clinical indices, then explored its feasibility and verified the performance.

## Materials and Methods

### Study Design

This nested case-control study aimed to determine whether the alternations in gut microbial composition during early pregnancy were associated with the later development of GDM, it was based upon an early pregnancy follow-up cohort. The cohort was established in the Hunan Provincial Maternal and Child Health Hospital (HPMCHH) in South China, from Mar 2017 to 2018 (ChiCTR1900020652). All the eligible participants provided written informed consent, and the study protocol was approved on Jan 11, 2017, by the Medical Ethical Committee of HPMCHH (EC201624). Details concerning inclusion criteria, sampling, questionnaires, anthropometrics, and biochemistry are provided as supplementary material (Supplementary Text 1). Participants were recruited in the first trimester (10–14 weeks) and followed up to 42 days postpartum. GDM was defined at 24–28 gestational weeks, using established criteria from the International Association of Diabetes and Pregnancy Study Groups (IADPSG) based on the results of a standard 2 h, 75-g oral glucose tolerance test (OGTT) (ADA, 2012). Pregnant women were diagnosed with GDM if one or more of following applied glucose levels were elevated: fasting ≥ 5.1 mmol/L, 1 h ≥ 10.0 mmol/L, 2 h ≥ 8.5 mmol/L (ADA, 2012). Controls with normal blood glucose throughout the pregnancy were recruited from the same cohort and matched 1:1 to the confirmed GDM cases with respect to age (±3 years), gestational age (±1 week), and sample collection date (±1 month). Considering the fluctuations in the results of single blood glucose values (Bonongwe et al., 2015), it was necessary to consolidate the definition of normoglycemia to ensure that the included controls were true non-cases. OGTT results of all the controls adhered to the standards proposed by the American College of Obstetricians and Gynecologists for the management of blood glucose during pregnancy (requiring fasting, 1, 2 h blood glucose values to be lower than 5.0, 7.8, 6.7 mmol/L, respectively) (Committee on Practice Bulletins-Obstetrics, 2013).

Excluding those who were lost to follow-up (abortion, induced labor, transfer, or reluctance to continue to cooperate), GDM was diagnosed in 112 of 828 women according to the IADPSG criteria. Of which, 98 cases with complete data and early pregnancy samples were included and matched to the same number of controls. According to the study design (Supplementary Figure 1), 70 cases and 70 controls (unmatched) were randomly selected as a discovery set to analyze differences between GDM cases and controls in operational taxonomic units (OTUs), taxonomy and function levels, and to explore the correlation between differential genera and clinical indices/differential functions. Pattern recognition analysis based on data of the discovery set was performed for the early identification model of GDM, and the performance of the model was verified using the external validation set consisting of the remaining 28 cases and 28 controls (unmatched).

### DNA Extraction and 16S rRNA Gene Amplicon Sequencing

All the stool samples were collected from the GDM cases and controls in the early pregnancy. Total fecal genomic DNA was extracted from about 180–200 mg feces using QIAamp Fast DNA Stool Mini Kit (Qiagen, Hilden, Germany), according to the manufacturer's instructions. Extracted DNA was stored at −20°C until used. The variable region V4 of the 16S rRNA gene was amplified using specific 515F and 806R primers with the barcodes (Kozich et al., 2013). All PCR reactions were carried out with Phusion High-Fidelity PCR Master Mix (New England Biolabs). PCR products were mixed at equidensity ratios and then purified with Qiagen Gel Extraction Kits (Qiagen, Germany). A sequencing library was generated using TruSeq DNA PCR-Free Sample Preparation Kit (Illumina, USA), then assessed on the Qubit^@^ 2.0 Fluorometer (Thermo Scientific) and Agilent Bioanalyzer 2100 system. The established library was sequenced on an Illumina HiSeq 2500 platform at Novogene Bioinformatics Technology Co., Ltd, and 250-bp paired-end reads were generated.

### Statistical Analysis

The 16S rRNA gene sequence data set were processed with QIIME2 version 2018.11 using default parameters (https://docs.qiime2.org/). DADA2 version 2018.4.0 (Callahan et al., 2016) was utilized to filter out noisy sequences, correct errors in marginal sequences, remove chimeric sequences, remove singletons, join denoised paired-end reads, and then to dereplicate those sequences into OTUs with 100% sequence similarity. Rarefaction curves were utilized to evaluate the effects of sequencing depth on the obtained number of OTUs. OTUs with a number of the sequences <0.005% of the total number of sequences were discarded as recommended (Navas-Molina et al., 2013). The resulting OTU table was used for downstream analysis. Taxonomy annotation was conducted with a Naive Bayes classifier trained on the SILVA database (version 132) (Quast et al., 2013), in order to define the features that best distinguish each taxonomic group. Sequences that were identified as members of Chloroplasts, Eukarya, Cyanobacteria, and Archaea were removed. Alpha diversity was calculated with QIIME2 software (version 2018.11) based on the sequence similarity at the 100% level, including index of Observed OTUs, abundance-based coverage estimator (ACE), Chao1 estimator, Shannon, Simpson, Heip's evenness measure (Heip_e) and Dominance. Beta diversity was measured by Jaccard, Bray-curtis, unweighted and weighted UniFrac distances (Lozupone and Knight, 2005). Principal coordinate analysis (PCoA) plots based on several distances were constructed to detect the differences in beta diversity, and PERMANOVA (permutated analysis of variance) was used to indicate differences in the overall microbial composition between the two groups (Lozupone and Knight, 2005). The functional profile was predicted with Tax4Fun (Asshauer et al., 2015), an open-source R package for the output obtained from the SILVA dataset (version 132). Pattern recognition analysis based on forward feature selection combined with linear discriminant analysis (LDA) was performed using R version 3.5.1 (RCT, 2019).

Normal distributed continuous variables were reported as mean ± standard deviation and analyzed using the paired *t*-test or two-sample *t*-test, while non-normal distributed continuous variables were reported as median with interquartile ranges (Q1–Q3) and analyzed using the Wilcoxon signed rank test or Mann-Whitney U test. For dichotomous variables, the McNemar chi-square test, Pearson's chi-square test or Fisher's exact test was applied. LEfSe (linear discriminant analysis effect size; Segata et al., 2011) with *P*-value cutoff 0.05 and LDA score cutoff 2 was utilized to obtain the differential taxa and functions between the two groups. For correlation analysis, Spearman's rank correlation was used. The microbiota-microbiota correlation network was constructed using SparCC algorithm (Friedman and Alm, 2012) and visualized with Cytoscape version 3.4.0 (Shannon et al., 2003). The predictive model was established as follows: First, the performance of each variable was evaluated on the training samples using Leave-one-out cross-validation, and the one with the best performance was selected into the model. Then, the remaining variables were added into the model sequentially. In each round, the performance of each variable combined with the selected ones were evaluated, and the one with the best performance was added into the model. The model with the best performance was finally utilized in this study. All statistical analyses were conducted with SPSS version 23.0 (SPSS Inc., Chicago, IL, USA) and R version 3.5.1, *P* < 0.05 was considered significant.

## Results

### Characteristics of the Participants

The characteristics of the study population are presented in Table 1. As expected, markers of body mass index (BMI), waist, glucose, insulin homeostasis, and high-sensitivity C-reactive protein were higher in GDM groups compared to the groups of normoglycemic women in early pregnancy. For all participants, women diagnosed with GDM had higher systolic pressure (SBP, *P* = 0.001) and diastolic pressure (DBP, *P* = 0.001), whereas the two groups were comparable in Edinburgh Postnatal Depression Scale score (*P* = 0.738) and daily average intake of all major types of food. About 20% (40/196) of the participants had supplemented yogurt, however, there was no significant difference in daily average yogurt intake between controls and women who developed GDM later (*P* = 0.711), and no other types of probiotic supplements were involved. Considering the test efficiency caused by sample size, the differences in trends for each index between controls and women who developed GDM later were similar in both the discovery set and the validation set. Except for the lower levels of aspartate aminotransferase (*P* = 0.047) and urea (*P* = 0.017) found in the discovery set, other characteristics remained comparable across the two data sets.

**Table 1:** Characteristics of the study population^#^.

	**All participants**			**Discovery set**			**Validation set**			P^*^
	**Controls (*N* = 98)**	**GDM (*N* = 98)**	***P***	**Controls (*N* = 70)**	**GDM (*N* = 70)**	***P***	**Controls (*N* = 28)**	**GDM (*N* = 28)**	***P***	
**Basic characteristics**										
Age (year)	31.5 (28.75–35)	31.0 (28.8–35.0)	0.207	32.5 (29.0–35.0)	31 (28.8–34.0)	0.314	29.0 (28.0–34.0)	33.0 (28.3–36.0)	0.099	0.879
Gravidity	2 (1–3)	2 (1–3)	0.597	2 (1–3)	2 (1–3)	0.998	2 (1–3)	2 (2–3)	0.147	0.725
Parity	1 (0–1)	1 (0–1)	0.873	1 (0–1)	1 (0–1)	0.355	0 (0–1)	1 (0–1)	0.109	0.602
PCOS, n (%)	6 (6.1%)	9 (9.2%)	0.607	5 (7.1%)	8 (11.4%)	0.382	1 (3.6%)	1 (3.6%)	1.000	0.288
Smoking history, n (%)	2 (2.0%)	2 (2.0%)	1.000	1 (1.4%)	2 (2.9%)	1.000	1 (3.6%)	0 (0%)	1.000	1.000
Drink history, n (%)	3 (3.1%)	3 (3.1%)	1.000	3 (4.3%)	1 (1.4%)	0.612	0 (0%)	2 (7.1%)	0.471	1.000
**Anthropometrics and behavioral factors in early pregnancy**										
Gestational age of recruit (weeks)	12.71 ± 0.76	12.65 ± 0.78	0.574	12.70 (12.25–13.30)	12.70 (12.10–13.10)	0.570	12.95 (12.33–13.08)	12.7 (12.3–13.25)	0.954	0.650
BMI	20.83 ± 2.65	22.79 ± 3.01	<0.001	21.22 ± 2.71	22.75 ± 3.16	0.003	19.86 (18.49–20.51)	23.28 (20.85–24.9)	<0.001	0.196
Waist (cm)	77.29 ± 7.33	81.53 ± 9.15	0.001	76.00 (73.00–81.00)	80.50 (75.75–88.00)	0.015	75.38 ± 7.78	81.96 ± 5.96	0.001	0.699
SBP (mmHg)	114.82 ± 9.57	119.17 ± 10.09	0.001	114.86 ± 9.41	119.71 ± 9.68	0.003	114.71 ± 10.13	117.82 ± 11.11	0.279	0.523
DBP (mmHg)	74.30 ± 8.39	76.87 ± 8.61	0.035	74.00 (66.00–82.00)	78.00 (72.50–83.00)	0.012	76.32 ± 8.25	76.54 ± 8.44	0.924	0.406
EPDS	8.48 ± 3.83	8.32 ± 3.15	0.738	8 (6–11)	9 (5–11)	0.973	8 (6–11)	7.5 (6–10)	0.644	0.517
Daily cereal intake (g)	150 (120–225)	150 (120–225)	0.941	150 (120–225)	150 (120–225)	0.907	150 (120–225)	150 (120–225)	0.993	0.240
Daily tuber intake (g)	14.3 (5.6–28.6)	14.3 (3.3–28.6)	0.494	13.81 (5–28.57)	14.29 (3.33–28.57)	0.884	20.71 (6.79–28.57)	12.14 (0.42–19.82)	0.154	0.971
Daily vegetable intake (g)	200.0 (142.5–400.0)	200.0 (120.0–400.0)	0.760	200 (120–400)	200 (120–300)	0.296	200 (200–400)	220 (170–400)	0.672	0.277
Daily fruit intake (g)	400.0 (200.0–450.0)	400.0 (200.0–400.0)	0.849	400 (200–412.5)	400 (200–400)	0.863	400 (200–562.5)	400 (200–400)	0.810	0.701
Daily meat intake (g)	40.0 (13.9–60.0)	37.1 (8.6–65.0)	0.987	40 (11.14–60)	37.14 (8.57–60)	0.943	60 (19.29–115)	35 (8.93–120)	0.552	0.195
Daily seafood intake (g)	0 (0–4.6)	0 (0–7.0)	0.590	0 (0–4.63)	1 (0–7.14)	0.429	0 (0–7)	0 (0–7.5)	0.861	0.689
Daily fresh water products intake (g)	8.0 (2.0–17.1)	8.6 (2.0–25.7)	0.397	8 (2.5–17.14)	8 (0.75–17.14)	0.650	6.29 (0–11.86)	17.14 (3.14–28.93)	0.050	0.505
Daily eggs intake (g)	50.0 (14.3–50.0)	50.0 (21.4–50.0)	0.437	42.86 (14.29–50)	42.86 (21.43–50)	0.537	50 (14.29–50)	50 (21.43–50)	0.657	0.992
Daily milk intake (g)	112.8 ± 97.9	132.3 ± 131.4	0.273	92.86 (24.46–178.93)	107.14 (55.71–200)	0.096	125.36 (37.5–237.5)	50.71 (2.5–190.89)	0.123	0.533
Daily beans intake (g)	15.0 (4.3–32.1)	18.6 (6.3–32.7)	0.730	21.43 (4.82–33.04)	21.43 (7.14–40.71)	0.696	10.71 (3.14–27.86)	11.07 (5.18–21.43)	0.811	0.102
Daily nuts intake (g)	21.4 (7.1–50.0)	38.6 (10.7–65.7)	0.053	21.43 (4.82–50)	38.57 (13.39–70.36)	0.045	26.79 (13.21–50)	40 (10.71–61.07)	0.468	0.639
Daily oil intake (g)	21.0 (21.0–24.0)	22.5 (21.0–24.0)	0.198	21.75 (21–24)	22.5 (21–24)	0.303	21 (21–24)	22.5 (21–24)	0.240	0.537
Daily salt intake (g)	10.5 (10.5–12.0)	10.5 (10.5–12.0)	0.259	10.5 (10.5–12)	10.5 (10.5–12)	0.375	10.5 (10.5–12)	10.5 (10.5–12)	0.332	0.599
Daily water intake (g)	1,000 (675–1,500)	1,025 (1000–1,500)	0.061	1,000 (737.5–1,500)	1,000 (875–1,425)	0.468	1,000 (600–1,150)	1,200 (1,000–1,575)	0.001	0.470
Daily yogurt intake (g)	0 (0–4.47)	0 (0–0)	0.711	0 (0–28.57)	0 (0–0)	0.431	0 (0–0)	0 (0–0)	0.439	0.383
**Biochemical characteristics in early pregnancy**										
Gestational age of blood sample (weeks)	12.83 ± 0.77	12.73 ± 0.92	0.341	12.84 ± 0.74	12.69 ± 0.83	0.265	12.81 ± 0.85	12.81 ± 1.14	0.998	0.891
HGB (g/L)	123.24 ± 9.27	126.99 ± 8.4	0.004	124 (115–130)	128 (120.75–132)	0.023	125 (119–127)	128 (121.25–134.75)	0.102	0.359
TG (mmol/L)	1.32 (1.08–1.59)	1.57 (1.22–2.03)	0.005	1.33 (1.08–1.72)	1.61 (1.2–2.12)	0.013	1.31 (1.08–1.57)	1.55 (1.25–1.77)	0.114	0.609
TCHOL (mmol/L)	4.49 ± 0.7	4.58 ± 0.73	0.400	4.49 ± 0.75	4.64 ± 0.73	0.237	4.42 (4.02–4.99)	4.37 (3.9–4.91)	0.682	0.355
HDLCH (mmol/L)	1.92 ± 0.4	1.8 ± 0.41	0.026	1.87 (1.67–2.11)	1.72 (1.47–2.11)	0.053	1.91 ± 0.46	1.77 ± 0.37	0.225	0.682
LDLCH (mmol/L)	2.36 ± 0.64	2.5 ± 0.65	0.116	2.37 ± 0.66	2.53 ± 0.64	0.140	2.31 ± 0.6	2.4 ± 0.67	0.622	0.237
ALB (g/L)	45.40 (43.80–47.20)	44.90 (42.68–46.93)	0.096	45.5 (43.8–47.1)	44.85 (42.93–47.3)	0.447	45.69 ± 3.06	44.64 ± 2.68	0.180	0.828
ALT (U/L)	12.55 (9.45–20.80)	16.15 (11.50–25.33)	0.005	12.65 (9.03–20.05)	15.3 (10.98–23.18)	0.077	12.25 (9.53–22.1)	18 (13.1–36.95)	0.007	0.164
AST (U/L)	17.00 (14.48–19.80)	17.15 (15–21.53)	0.208	16.65 (14.08–20.18)	16.8 (14.93–20.83)	0.648	17.3 (16.03–19.3)	18.5 (15.43–25.43)	0.314	0.047
GGT (U/L)	10.0 (8.0–15.0)	12.0 (9.0–19.0)	0.017	10.5 (8.75–16)	12 (9–19)	0.174	9.5 (7–14.75)	12 (8.25–18.75)	0.181	0.417
CREA (umol/L)	43.01 ± 5.75	43.09 ± 5.5	0.923	42.5 (38.75–46)	43 (39.75–46.4)	0.491	44 (41.25–47)	43 (40.5–46)	0.588	0.219
UA (umol/L)	193.05 ± 42.98	216.22 ± 47.94	0.001	190.24 ± 42.53	214.90 ± 47.82	0.002	200.07 ± 44.07	219.54 ± 48.93	0.124	0.386
UREA (umol/L)	2.59 ± 0.62	2.62 ± 0.70	0.786	2.5 (2.1–3)	2.4 (2–3.03)	0.828	2.75 (2.15–3.1)	2.8 (2.4–3.1)	0.812	0.017
FT4 (pmol/L)	16.85 ± 2.90	16.40 ± 2.45	0.254	16.33 (15.12–18.15)	15.94 (14.69–17.4)	0.400	16.99 (15.37–18.13)	16.87 (15.11–18.07)	0.635	0.231
TSH (mIU/L)	1.14 (0.53–2.01)	1.43 (0.82–2.15)	0.194	1.16 (0.56–2.02)	1.53 (0.8–2.21)	0.110	1.07 (0.5–1.94)	1.34 (0.84–1.71)	0.528	0.369
TPOAB (IU/mL)	12.21 (9.66–18.02)	13.41 (10.35–17.07)	0.774	12.61 (9.79–17.41)	13.02 (10.08–16.74)	0.772	11.7 (9.55–18.99)	14.01 (11.3–19.47)	0.077	0.828
FGB (mmol/L)	4.63 (4.40–4.74)	4.89 (4.56–5.16)	<0.001	4.62 (4.3–4.74)	4.82 (4.54–5.15)	<0.001	4.65 (4.51–4.84)	4.91 (4.6–5.19)	0.042	0.466
Fasting insulin (pmol/L)	46.46 (28.47–70.42)	64.03 (44.24–90.77)	0.010	48.06 (30.97–72.09)	63.41 (44.45–90.77)	0.018	40.28 (25.00–66.32)	68.41 (39.38–100.7)	0.025	0.649
Leptin (ng/mL)	6.20 (4.54–8.85)	6.25 (4.89–9.41)	0.393	6.3 (4.76–9.2)	6.43 (4.89–9.51)	0.778	5.73 (4.06–6.94)	5.45 (4.83–9.15)	0.706	0.164
Adiponectin (ng/mL)	22.25 (11.95–41.46)	27.93 (13.09–44.09)	0.080	25.88 (14.61–43.13)	29.63 (11.6–47.13)	0.683	17.62 (7.77–34.34)	25 (14.76–40.04)	0.216	0.136
hsCRP (mg/L)	1.61 (0.89–3.03)	2.57 (1.65–4.18)	<0.001	1.69 (0.97–3.79)	2.43 (1.55–4.37)	0.024	1.3 (0.83–2.01)	3.04 (1.84–4.14)	0.001	0.505

# *Normal distributed continuous variables were reported as mean ± standard deviation and analyzed using the paired t-test or two-sample t-test, while non-normal distributed continuous variables were reported as median with interquartile ranges (Q1–Q3) and analyzed using the Wilcoxon signed rank test or Mann-Whitney U-test. For dichotomous variables, the McNemar chi-square test, Pearson's chi-square test or Fisher's exact test was applied*.

* *Comparison was conducted between the discovery set (n = 140) and validation set (n = 56)*.

*ALB, albumin; ALT, alanine aminotransferase; AST, aspartate aminotransferase; BMI, body mass index; CREA, creatinine; DBP, diastolic pressure; EPDS, edinburgh postnatal depression scale; FBG, fasting blood glucose; FT4, free thyroxine; GDM, gestational diabetes mellitus; GGT, glutamine transpeptidase; GH, gestational hypertension; HDLCH, high density cholesterol; HGB, hemoglobin; hsCRP, high-sensitivity C-reactive protein; LDLCH, low density cholesterol; MET, metabolic equivalent of task; PCOS, polycystic ovarian syndrome; SBP, systolic pressure; TCHOL, total cholesterol; TG, triglycerides; TPOAB, thyroid peroxidase antibody; TSH, thyroid stimulating hormone; UA, uric acid; UREA, urea*.

### Altered Gut Microbiota in Women Who Developed GDM Later

**Figure 1:**
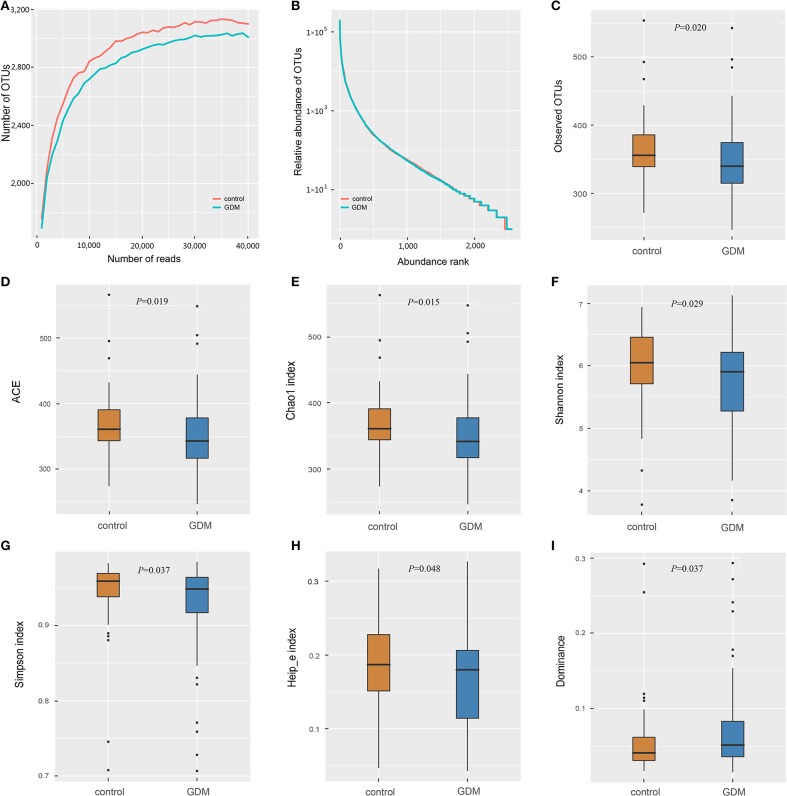
The richness and diversity of the gut microbiota in healthy control and GDM groups. **(A)** Rarefaction curves to estimate the richness of the gut microbiota in healthy control and GDM groups. The vertical axis shows the number of OTUs expected after sampling the number of tags or sequences shown on the horizontal axis. **(B)** Rank abundance curves of bacterial OTUs derived from the two groups. Alpha diversity was calculated with QIIME2 software (version 2018.11) based on the sequence similarity at the 100% level, including index of Observed OTUs **(C)**, abundance-based coverage estimator (ACE, **D**), Chao1 estimator **(E)**, Shannon **(F)**, Simpson **(G)**, Heip's evenness measure (Heip_e, **H**) and Dominance **(I)**. Unpaired *t*-tests (two-tailed) were used to analyze variation among the two groups, and the *P*-values are shown in the corresponding figures.

The 16S rRNA gene sequencing of the stool samples collected from controls and women who developed GDM later yielded 12,390,684 high-quality reads, with an average of 63,218 reads per sample. Of which, the discovery set comprised 8,940,122 high-quality reads (~63,858 reads per sample; Supplementary Table 1). Rarefaction curves (Figure 1A) indicated that the number of OTUs tended to be stable when the number of reads exceeded 30,000. Thus, 36,000 sequences were randomly selected from each sample to normalize the data. The abundance rank curves (Figure 1B) showed that species uniformity was similar between the two groups in the discovery set. The analysis of alpha diversity showed that the women who developed GDM later presented lower richness (lower Observed OTUs, *P* = 0.020; ACE, *P* = 0.019; Chao1, *P* = 0.015) and also lower diversity (lower Shannon, *P* = 0.029; Simpson, *P* = 0.037; Heip_e, *P* = 0.048; and higher Dominance, *P* = 0.037) than that detected in the controls (Figures 1C–I). PCoA plots based on several distances were constructed to detect the differences in beta diversity, and PERMANOVA analysis indicated differences in the overall microbial composition between the two groups (Supplementary Figure 2).

**Figure 2:**
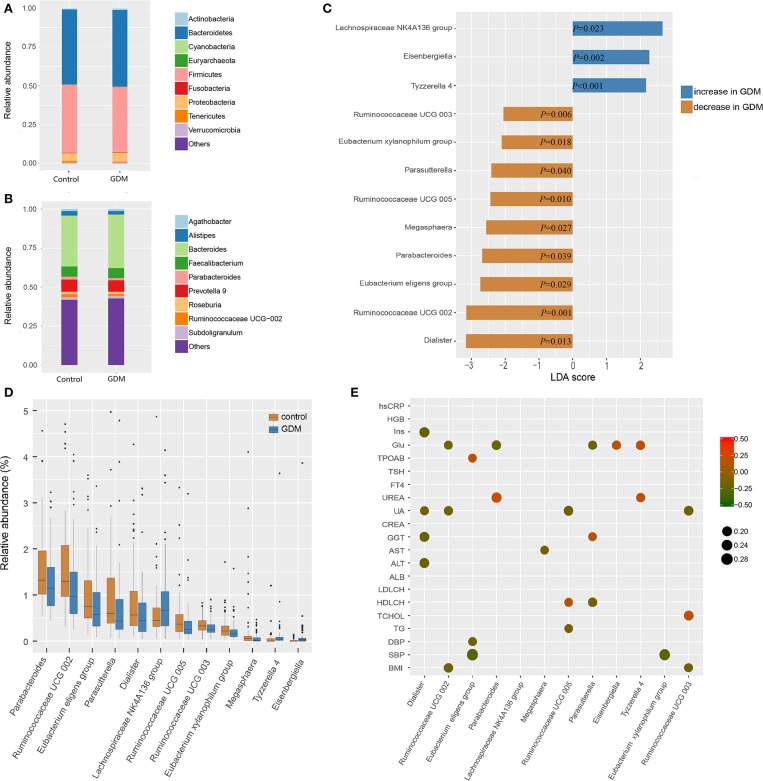
Different bacterial taxa between healthy control and GDM groups. **(A)** Relative abundance of the top 10 bacterial taxa at the level of bacterial phylum. **(B)** Relative abundance of the top 10 bacterial taxa at the level of bacterial genus. **(C)** Linear discriminant analysis (LDA) score of differential taxa at genus level based on LEfSe (*P* < 0.05 and LDA threshold value >2). The abscissa is the LDA value, the GDM-enriched genera are shown as blue bars, the control-enriched genera are shown as orange bars, and the *P*-value is shown in each bar. **(D)** Boxplot of differential taxa at genus level. **(E)** Spearman's rank correlation heatmap of differential taxa at genus level and clinical indices. Circles that tend to red and tend to green represent positive and negative correlations, respectively.

The top 10 phyla and 10 genera of gut microbiota for the discovery set are shown in Figures 2A,B. Notably, the gut microbiota was dominated by Bacteroidetes, Firmicutes and Proteobacteria in descending order. The predominant genus found in both groups was *Bacteroides*, which was increased in the GDM group, but without statistical significance (*P* = 0.550). To further explore the altered gut microbiota in women who developed GDM later, we compared bacterial abundance between groups at the levels of phylum, family, and genus. As shown in Figures 2C,D, at the genus level, women who developed GDM later showed a significantly higher abundance of *Eisenbergiella* (*P* = 0.002), *Tyzzerella 4*, (*P* < 0.001) and *Lachnospiraceae NK4A136* group (*P* = 0.023), while the healthy controls were significantly enriched for *Parasutterella* (*P* = 0.040), *Parabacteroides* (*P* = 0.039), *Megasphaera* (*P* = 0.027), *Dialister* (*P* = 0.013), *Ruminococcaceae UCG 005* (*P* = 0.010), *Ruminococcaceae UCG 002* (*P* = 0.001), *Ruminococcaceae UCG 003* (*P* = 0.006)*, Eubacterium xylanophilum* group, (*P* = 0.018), and *Eubacterium eligens* group (*P* = 0.029). Relative abundance of the differential genera is presented in Supplementary Table 2, while the statistical parameters and LDA scores are presented in Supplementary Table 3. These findings revealed dysbiosis of the gut microbiota among women who developed GDM later.

In order to explore potential clinical paths by which changes in maternal gut microbiota might lead to GDM, we investigated whether differential taxa were associated with maternal clinical indices in the early pregnancy (Figure 2E and Supplementary Tables 4, 5). We observed that GDM-enriched genera *Eisenbergiella* (*P* = 0.029, *r* = 0.185) and *Tyzzerella 4* (*P* = 0.022, *r* = 0.194) were positively correlated with fasting blood glucose levels, while three control-enriched genera: *Parabacteroides* (*P* = 0.018, *r* = −0.200), *Parasutterella* (*P* = 0.036, *r* = −0.177) and *Ruminococcaceae UCG 002* (*P* = 0.044, *r* = −0.171) were negatively correlated with fasting blood glucose levels. Furthermore, the control-enriched genus *Dialister* was negatively correlated with fasting insulin (*P* = 0.006, *r* = −0.232), daily oil (*P* = 0.030, *r* = −0.184) and yogurt intake (*P* = 0.025, *r* = −0.189). These results indicated that the collective expression of these bacteria could be associated with the subsequent development of GDM.

### Inferred Functional Changes in the Gut Microbiota of Women Who Developed GDM Later

**Figure 3:**
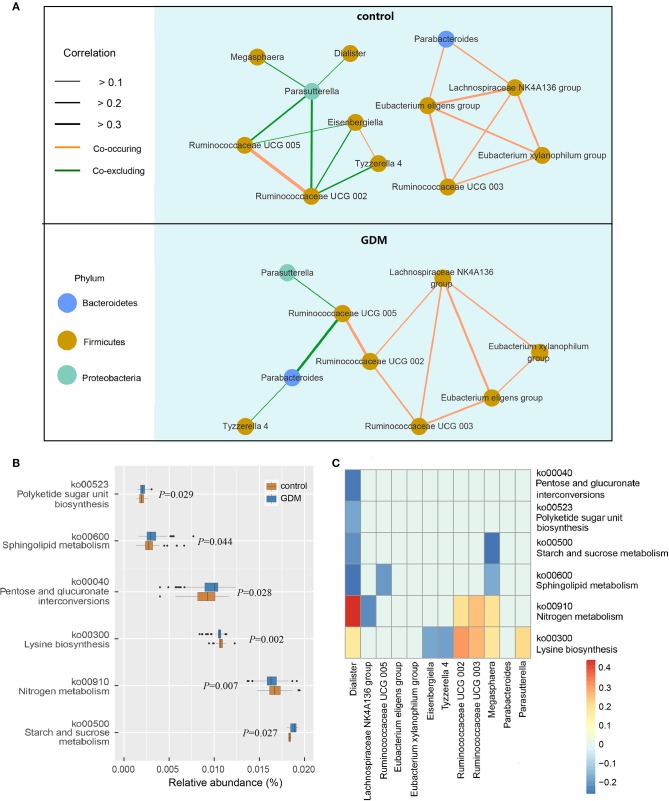
Functional analysis of the gut microbiota in healthy control and GDM groups. **(A)** Co-occuring and co-excluding networks for differential genera in control and GDM groups (yellow line: co-occuring; green line: co-excluding). **(B)** Boxplot of differential functions between control and GDM groups. The differential pathways were identified using Tax4Fun and LEfSe, the *P*-values next to each boxplot. **(C)** Heatmap of Spearman's rank correlations between differential genera (LEfSe: *P* < 0.05 and linear discriminant analysis threshold value >2) and differential pathways. Bars that tend to red and tend to blue represent positive and negative correlations, respectively.

The network of interactions between gut microbiota may differ in various health states. Thus, we explored the co-occurring and co-excluding networks of differential genera for healthy controls and women who developed GDM later. As shown in Figure 3A, a highly complex network of interactions was found in the control group encompassing 12 differential genera, conversely, networks were limited in the GDM group with correlations related to genera of *Dialister, Megasphaera, Eisenbergiella* no longer significant. Within the Firmicutes phylum, the positive association between genera of *Ruminococcaceae UCG 003* and *Eubacterium xylanophilum* group disappeared in GDM group, and were replaced with new positive correlations between *Ruminococcaceae UCG 002* and *Ruminococcaceae UCG 003*, as well as *Lachnospiraceae NK4A136* group. Between Bacteroidetes and Firmicutes phyla, the strong negative relationship between *Parasutterella* and *Ruminococcaceae UCG 002* in the control group disappeared in the GDM group. Meanwhile, the positive correlations between Proteobacteria and Firmicutes phyla in the control group turned to negative in the GDM group.

Using Tax4Fun and LEfSe we identified 6 differential pathways between controls and women who developed GDM later (Figure 3B), the predicted metagenomes for women who developed GDM later depicted an enrichment of polyketide sugar unit biosynthesis (ko00523, *P* = 0.029), sphingolipid metabolism (ko00600, *P* = 0.044), pentose and glucuronate interconversions (ko00040, *P* = 0.028), starch and sucrose metabolism (ko00500, *P* = 0.027), as well as a reduction in lysine biosynthesis (ko00300, *P* = 0.002) and nitrogen metabolism (ko00910, *P* = 0.007). In the process of plotting correlations between differential genera and inferred metabolic pathways (Figure 3C and Supplementary Table 6), we observed significant positive relationships between several control-enriched genera (*Dialister, Ruminococcaceae UCG 002, Ruminococcaceae UCG 003, Megasphaera, Parasutterella*) and control-enriched pathways (ko00300, ko00910). Negative correlations were observed between GDM-enriched genera and control-enriched pathways, as well as between control-enriched genera and GDM-enriched pathways. Moreover, Spearman's rank correlation analyses indicated that starch and sucrose metabolism pathway was positively associated with fasting blood glucose (*P* = 0.022, *r* = 0.194) and total cholesterol (*P* = 0.023, *r* = 0.193), lysine biosynthesis pathway was negatively associated with fasting blood glucose (*P* = 0.033, *r* = −0.181) and leptin (*P* = 0.043, *r* = −0.171). Although the pathway analyses are predictive, they indicated that impairment of gut microbiota may contribute to the onset of GDM through the interference of physiological metabolic functions.

### Gut Microbiota-Based Pattern Recognition Analysis for the Diagnosis of GDM

**Figure 4:**
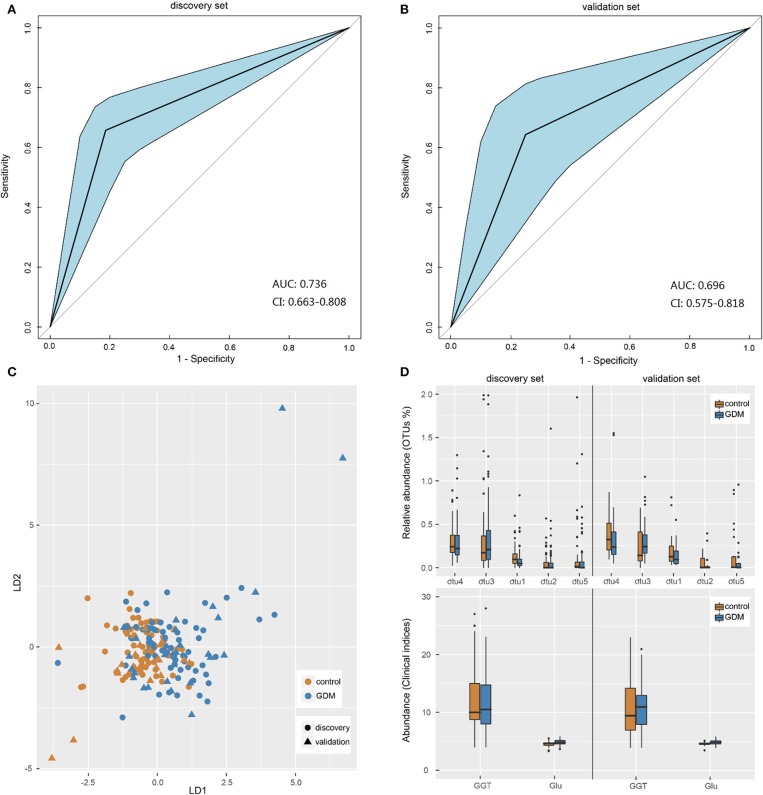
Gut microbiota-based pattern recognition analysis for the diagnosis of GDM. **(A,B)** Receiver operating characteristic (ROC) curves for samples in discovery and validation sets. AUC, the areas under ROC curves; CI, confidence interval. **(C)** Scatter plot of all samples in discovery and validation sets based on first two axes obtained from Linear discriminant analysis. **(D)** The boxplot of the seven features utilized in the model for samples in discovery and validation sets. The otu1, 2, 3, 4, 5 were genera *Uncultured-Ruminococcaceae, Alloprevotella, Ruminococcus 2, Parabacteroides*, and *Ruminococcaceae UCG-014*, respectively. GGT, glutamine transpeptidase; Glu, fasting blood glucose.

Pattern recognition analysis was performed to assess the predicative ability of gut microbiota and clinical data for GDM status. An LDA model was established utilizing a set of 5 OTUs (*Parabacteroides, Ruminococcus 2, Ruminococcaceae UCG-014, Alloprevotella*, and *uncultured-Ruminococcaceae*) and 2 clinical indices (GLU, GGT). The receiver operating characteristic (ROC) curves demonstrated that the model showed accuracy and efficacy in the identification of GDM patients in early pregnancy, with the areas under ROC curves (AUC) of the discovery (Figure 4A) and validation (Figure 4B) sets being 0.736 (95% confidence interval: 0.663–0.808) and 0.696 (95% confidence interval: 0.575–0.818), respectively. In the new dimension defined by the model variables, samples in different groups could be effectively distinguished (Figure 4C). Moreover, distribution trends of the 7 categorical indices between the two groups were similar in both data sets (Figure 4D), the relative abundance of genus *uncultured-Ruminococcaceae* was significantly higher in the controls both in the discovery (*P* = 0.001) and validation (*P* = 0.029) sets, while the level of fasting GLU was significantly higher in GDM group both in the discovery (*P* < 0.001) and validation (*P* = 0.042) sets. Performance of LDA models (Supplementary Table 7) suggested that the combined model outperformed models based on five bacterial markers or two clinical indices alone, with higher accuracy and specificity both in the discovery set and validation set. Thus, a similar model could be utilized as a new technology for the early detection of GDM patients.

## Discussion

### Principal Findings

In the present study, we sequenced the 16S rRNA gene for total bacterial DNA of stool samples from 98 GDM and 98 matched healthy controls in their early pregnancy (10–15 weeks). The results demonstrated that aberrant gut microbiota interactions were associated with GDM before its onset, which was mainly reflected through the observed alterations in gut microbial composition and bacterial gene functions. The prediction model with external validation also demonstrated the potential and feasibility of utilizing gut microbiota as a non-invasive biomarker for the diagnosis of GDM. This study will provide a better understanding of the association between gut microbiota and GDM. Ultimately, it will help identify novel microbiota-related diagnostic tools and preventive strategies.

### Alterations in Gut Microbial Composition and Functional Annotation Precede the Onset of GDM

The data set was randomly split into a discovery set and a validation set. Based on bioinformatic analyses of the discovery set, decreased richness (alpha diversity) and increased individual diversity (beta diversity) were observed in women who would eventually develop GDM. Specifically, *Eisenbergiella, Tyzzerella 4*, and *Lachnospiraceae NK4A136* group were enriched in women with subsequent GDM. Moreover, these GDM-enriched bacteria were reported to have been involved in gut bacterial dysbiosis in previous studies (Kelly et al., 2016; Zhao et al., 2017; Crusell et al., 2018). *Eisenbergiella* was recently reported to be associated with higher gestational weight gain (Crusell et al., 2018). *Tyzzerella 4* were potentially pathogenic bacteria linked to increased cardiovascular disease risk (Kelly et al., 2016). Moreover, both *Eisenbergiella* and *Tyzzerella 4* were positively correlated with fasting blood glucose levels, suggesting that they might have direct associations with GDM pathophysiology. *Lachnospiraceae NK4A136* group were previously reported in several animal studies with various conclusions. Zhao et al. (2017) considered *Lachnospiraceae NK4A136* group to be intestinal probiotics showing a negative correlation to intestinal inflammation in rats; while Zheng et al. (2018) suggested that the *Lachnospiraceae NK4A136* group was an indicator of gut dysbiosis, higher relative abundances of *Lachnospiraceae NK4A136* group were found in rats with advanced-stage type 1 diabetes (Gao et al., 2019).

Meanwhile, the microbes reduced in women with subsequent GDM included *Parasutterella, Parabacteroides, Megasphaera, Dialister, Ruminococcaceae UCG 005, Ruminococcaceae UCG 002, Ruminococcaceae UCG 003, Eubacterium xylanophilum* group, and *Eubacterium eligens* group. Most importantly, the pronounced reduction of *Parabacteroides, Dialister*, and *Eubacterium eligens* group had been previously observed in diagnosed GDM patients in comparison to healthy pregnant women (Kuang et al., 2017; Cortez et al., 2018), suggesting that changes in these bacteria were closely related to GDM, and occurred over time prior to the development of GDM, the potential pathological mechanism is worthy of further research utilizing sterile animal verification trials. Most of these bacteria, including *Parabacteroides, Megasphaera, Ruminococcaceae UCG 005, Ruminococcaceae UCG 002, Eubacterium xylanophilum* group and *Eubacterium eligens* group, could produce short-chain fatty acids (SCFAs) such as butyrate, propionate, and acetate (Chen et al., 2017; Chung et al., 2017; Gao et al., 2018; Wang et al., 2018b, 2019; Metzler-Zebeli et al., 2019), which could maintain normal physiological functioning of the intestines, regulate gut permeability, increase insulin sensitivity, and induce gut inflammatory responses that recede the development of diabetes (Vaarala et al., 2008). Additionally, the study provided a clear insight into the correlations between differential genera and maternal clinical indices, besides several reported associations (Guo et al., 2018; Wei et al., 2018), the others, especially the associations with blood pressure, blood lipids, dietary intake, liver and kidney functions, still need validation via continued research in this field. Moreover, studies regarding the potential causal links between the collective effects of gut microbiota and their relationship to GDM are also warranted.

Concomitant with the alteration of gut microbial composition, we observed changes in bacterial gene functions. Functional annotation indicated that a significant decrease in the lysine biosynthetic pathway was related to GDM. As an essential amino acid required in protein synthesis, lysine is also used in the peptidoglycan layer of Gram-positive bacterial cell walls, the lysine biosynthetic pathway offers several potential antibacterial enzyme targets, highlighting their importance for bacterial cell survival and the healthy homeostasis of the gut microbiota (Gillner et al., 2013). In contrast, enrichment of modules for sphingolipid metabolism in the GDM group may hint at a potential role of gut microbiota in inflammatory signaling (Maceyka and Spiegel, 2014), as bacterial sphingolipid production resulted in changes in host sphingolipid metabolites (Brown et al., 2019), and sphingolipid metabolism was a converging point linking excess free fatty acids and inflammation aroused by adipose-derived inflammation (Kang et al., 2013). Moreover, sphingolipid metabolism also played a crucial role in glucolipotoxicity induced apoptosis and loss of function of pancreatic β cells, contributing to the progression of insulin resistance (Kang et al., 2013; Veret et al., 2014). Consistent with the findings in T2DM and obesity (Haus et al., 2009), the link between sphingolipids, inflammation, and insulin resistance was likely responsible for GDM pathology. Functional analysis in the present study also found two carbohydrate metabolism pathways (pentose and glucuronate interconversions, starch and sucrose metabolism) that were enriched in the GDM group, both of which were previously found to be associated with T2DM (Zhao et al., 2013; Sun et al., 2014), and might be used for GDM prevention.

### Feasibility of Microbial Biomarkers for the Early Prediction of GDM

Early prediction of GDM allows for potential implementation of interventions to reduce the risk of subsequent adverse maternal and fetal outcomes. Here, we constructed an LDA model to assess the predictive performances of gut microbiota for GDM, based on the fact that microbes within the human gut are important contributors to the host metabolism (Le Chatelier et al., 2013), and gut microbial communities could be modified by improving diet and lifestyle, or through intervention with probiotics (Ray, 2014; Homayouni et al., 2019). Five OTUs (*Parabacteroides, Ruminococcus 2, Ruminococcaceae UCG-014, Alloprevotella*, and *Uncultured-Ruminococcaceae*) and 2 clinical indices (GLU, GGT) were included in this model, with the AUC of the training and validation sets being 0.736 (0.663–0.808) and 0.696 (0.575–0.818), respectively, which outperformed models based on five bacteria markers or two clinical indices alone. Previous predictions of GDM were largely dependent on recognized risk factors, the most important of which include ethnicity, maternal age, BMI, family history of T2DM, and prior GDM (Kennelly and McAuliffe, 2016). Several risk-prediction tools (Naylor et al., 1997; van Leeuwen et al., 2010; Teede et al., 2011; Gabbay-Benziv et al., 2015) based exclusively on these clinical characteristics available in the early pregnancy have been proposed to identify women at high risk of developing GDM. External validation of four clinical risk-prediction models using our cohort yielded a lower performance than those observed in the original studies, mainly having an unsatisfactory AUC in the discovery set and over-fitting in the validation set (Supplementary Table 8). Despite the differences in characteristics of the population, diagnostic criteria used, and prevalence of GDM, the compared results might indicate that the LDA model utilizing both clinical and gut microbiota-targeted biomarkers demonstrated effective performance in the prediction of GDM. However, considering the strong associations between gut microbiota and host location (He et al., 2018), as well as ethnicity (Deschasaux et al., 2018), it is necessary to carry out further verification on other populations, to assess whether the gut microbiota as a predictor of GDM is applicable to people with different races and regions. The confirmed conclusion would provide a new direction for the early detection and prevention of GDM.

### Strengths and Limitations

This nested case-control study was based on a prospective follow-up cohort. Comparison of the gut microbial composition and function in general pregnant women before the onset of GDM provided a better understanding of the association between gut microbiota and GDM. Exploration of the prediction model provided a novel microbiota-related direction for the early detection of GDM. However, several limitations of this study need to be addressed, and thus, merit further discussion. First, the sample size was small, especially the validation set, and the performances of LDA models were not very satisfactory in external validation samples. Second, all of the participants were from the same research site, and only one fecal sample per participant was analyzed. Samples in this prospective study were collected in early pregnancy, but no fecal samples prior to pregnancy were available for comparison. Moreover, samples collected at a single time point would not convey the entire dynamic relationship. Third, the biochemical baselines with significant differences might have some confounding. Fourth, we had no information on the levels of immune inflammatory factors and intestinal metabolites that were working in correlation with the gut microbiota. In order to address these limitations and confirm the findings observed in the current study, a multi-center, multi-point, vertical cohort investigation with analyses of gut genome and metabolome will be required.

## Conclusion

In summary, we characterized aberrant gut microbiota found in early pregnancy in women who eventually developed GDM, and reported a diagnostic model protocol with external validation of microbial markers for GDM. If confirmed by further large-sampled well-designed studies, these results suggest that gut bacterial dysbiosis in early pregnancy might be involved in the pathogenesis of GDM, and gut microbiota-targeted biomarkers might be potential predictors of GDM. Elucidating these findings in the gut microbiota will provide a foundation to improve our understanding of GDM etiology and support potential preventive options based on modifying the gut microbiota.

## Data Availability Statement

The raw sequence data reported in this paper have been deposited in the Genome Sequence Archive in BIG Data Center, Beijing Institute of Genomics (BIG), Chinese Academy of Sciences, under accession numbers CRA001832, the shared URL is http://bigd.big.ac.cn/gsa/s/QP5ltTlv.

## Ethics Statement

The studies involving human participants were reviewed and approved by Medical Ethical Committee of Hunan Provincial Maternal and Child Health Hospital. The patients/participants provided their written informed consent to participate in this study. Written informed consent was obtained from the individual(s) for the publication of any potentially identifiable images or data included in this article.

## Author Contributions

HT and SM designed the study. SM, YY, LH, SL, JZ, CG, NZ, XW, and YX recruited participants, collected basic data and samples. SM, LH, and SL analyzed the data. HT, LH, SL, JZ, CG, NZ, XW, and YX contributed to discussion and reviewed/edited the manuscript. SM and YY wrote the manuscript. HT supervised the study and the guarantor of this work. All authors read and approved the final manuscript.

### Conflict of Interest

The authors declare that the research was conducted in the absence of any commercial or financial relationships that could be construed as a potential conflict of interest.
