# Supplementary material for: Alterations in Gut Microbiota of Gestational Diabetes Patients During the First Trimester of Pregnancy
Source: Front Cell Infect Microbiol. 2020 Feb 27;10:58. doi: 10.3389/fcimb.2020.00058 (PMC7056672; doi:10.3389/fcimb.2020.00058)
Supplement: Supplementary file 1 [file Data_Sheet_1.DOCX]

**Supplementary materials**

[Supplementary Text 1 2](#_Toc25314281)

[Supplementary Table 1 4](#_Toc25314282)

[Supplementary Table 2 9](#_Toc25314283)

[Supplementary Table 3. 14](#_Toc25314284)

[Supplementary Table 4. 15](#_Toc25314285)

[Supplementary Table 5 16](#_Toc25314286)

[Supplementary Table 6 17](#_Toc25314287)

[Supplementary Table 7 18](#_Toc25314288)

[Supplementary Table 8 19](#_Toc25314289)

[Supplementary Fig. 1 20](#_Toc25314290)

[Supplementary Fig. 2 21](#_Toc25314291)

# Supplementary Text 1

**Inclusion criteria**

Pregnant women were recruited in their first trimester (10-14 weeks) and followed up to 42 days postpartum. The detailed inclusion criteria were as follow: (i) singleton pregnancy and natural conception; (ii) diabetes-free at recruitment, without diseases that might affect microbiome composition or glucose metabolism, like pre-pregnancy diabetes, thyroid disorders, hypertension, inflammatory bowel disease and cardiovascular diseases; (iii) had not received any antibiotic treatment throughout the current pregnancy; (iv) no acute infection 2 weeks before sample collection; (v) planned to complete regular obstetric examinations and the final delivery at current hospital.

**Sampling**

Blood samples (3-5 mL) were collected in the early pregnancy (10-15 weeks of gestation) by certified nurses in the morning following a 10-h overnight fast. All the samples were stored at 4 °C immediately after collection and transported back to the laboratory on ice within 8 hours. Separated serum after centrifugation (3500 rpm for 15 min) were divided in three equal parts and stored at -80 °C until assay. All the participants were instructed on how to slef-collect the fecal samples, and the materials, including sterile container, glove and mask were provided in a convenient collection kit. Fecal sample (about 10 g) was freshly collected in sterile tube by each participant before 16 weeks of gestation and immediate freezing at -20 °C. The samples were transferred to the laboratory on dry ice every Friday and stored at -80 °C until DNA extraction. No storage medium was used.

**Questionnaires**

Participants completed interviewer- and self-administered, validated questionnaires on baseline characteristics and lifestyle, including information on age, gravidity, parity, polycystic ovarian syndrome (PCOS), GDM history, gestational hypertension history, smoking history, drink history, dietary intake, depression and physical activity. Of which, dietary intake was recorded using a frequency questionnaire of food groups; depression was assessed using the Edinburgh Postnatal Depression Scale (EPDS). All the questionnaires were completed by trained research staff in accordance with the instructions.

**Anthropometrics**

Anthropometric data were collected according to standard procedures. Body weight (kg) and height (m) were measured in light clothes and without shoes. Body mass index (BMI) was calculated by dividing the weight (kg) by the square of height (m). Waist circumference was measured midway between the iliac crest and the lower costal margin in a standing position. Systolic pressure (SBP) and diastolic pressure (DBP) were measured using a wrist electronic sphygmomanometer (Omron HEM-6050, Japan) in a sitting position after a 15 minutes rest.

**Biochemistry**

Information about routine biochemical indicators, such as hemoglobin (HGB), triglycerides (TG), total cholesterol (TCHOL), high density cholesterol (HDL), low density cholesterol (LDL), serum albumin (ALB), alanine aminotransferase (ALT), aspartate aminotransferase (AST), creatinine (CREA), uric acid (UA), urea (UREA), free thyroxine (FT4), thyroid stimulating hormone (TSH), thyroid peroxidase antibody (TPOAB), fasting blood glucose (FBG) and glucose tolerance test (OGTT) were extracted from clinical records of the Hospital Information Systems used in HPMCHH. Moreover, fasting insulin was measured on a Roche cobas e601 using an electrochemiluminescence immunoassay (Roche Diagnostics GmbH, Mannheim, Germany). Glutamine transpeptidase (GGT) and high-sensitivity C-reactive protein (hsCRP) were measured on a Roche cobas c501 system, using a rate method (Roche) and a particleenhanced turbidimetric immunoassay (Roche), respectively. Adiponectin and leptin were measured using enzyme linked immunosorbent assay (ELISA) kits (Shenzhen ziker Biological Technology Co., Ltd) on microplate reader (Autobio, China).

| Supplementary Table 1**. Data production of all the 196 samples** | | | | | |
| --- | --- | --- | --- | --- | --- |
| Sample-ID | Set | Input (reads) | Filtered (reads) | Denoised (reads) | Non-chimeric (reads) |
| c1 | discovery | 81349 | 70644 | 70644 | 69139 |
| c10 | discovery | 74965 | 61456 | 61456 | 59692 |
| c100 | discovery | 51003 | 40805 | 40805 | 40605 |
| c11 | discovery | 76616 | 65778 | 65778 | 63006 |
| c12 | discovery | 83951 | 71986 | 71986 | 70010 |
| c13 | discovery | 81209 | 67547 | 67547 | 63860 |
| c14 | discovery | 68252 | 56006 | 56006 | 54154 |
| c15 | discovery | 77625 | 67808 | 67808 | 65417 |
| c16 | discovery | 82947 | 68062 | 68062 | 66095 |
| c18 | discovery | 80590 | 69632 | 69632 | 67980 |
| c19 | discovery | 73182 | 65196 | 65196 | 60903 |
| c2 | discovery | 82757 | 73941 | 73941 | 67478 |
| c20 | discovery | 89749 | 78172 | 78172 | 75644 |
| c21 | discovery | 83358 | 72129 | 72129 | 70088 |
| c22 | discovery | 46108 | 37940 | 37940 | 37062 |
| c23 | discovery | 86050 | 76296 | 76296 | 74455 |
| c24 | discovery | 80821 | 69801 | 69801 | 67731 |
| c25 | discovery | 62613 | 51993 | 51993 | 50489 |
| c27 | discovery | 83899 | 72835 | 72835 | 70499 |
| c28 | discovery | 72295 | 64046 | 64046 | 62541 |
| c3 | discovery | 79580 | 71084 | 71084 | 67309 |
| c30 | discovery | 84636 | 73762 | 73762 | 71639 |
| c32 | discovery | 79039 | 68186 | 68186 | 62211 |
| c33 | discovery | 88442 | 71812 | 71812 | 71396 |
| c34 | discovery | 84530 | 68379 | 68379 | 66470 |
| c36 | discovery | 87949 | 71818 | 71818 | 71355 |
| c37 | discovery | 79512 | 70367 | 70367 | 69580 |
| c39 | discovery | 76517 | 63196 | 63196 | 60935 |
| c42 | discovery | 85561 | 76191 | 76191 | 75433 |
| c45 | discovery | 74847 | 60553 | 60553 | 59942 |
| c50 | discovery | 84265 | 68171 | 68171 | 67627 |
| c51 | discovery | 88567 | 73609 | 73609 | 72628 |
| c52 | discovery | 77971 | 62725 | 62725 | 62078 |
| c54 | discovery | 89809 | 79552 | 79552 | 77974 |
| c55 | discovery | 89256 | 82919 | 82919 | 81035 |
| c56 | discovery | 76471 | 68571 | 68571 | 67849 |
| c57 | discovery | 76452 | 67787 | 67787 | 66156 |
| c58 | discovery | 87289 | 77564 | 77564 | 74781 |
| c59 | discovery | 87943 | 78217 | 78217 | 76029 |
| c60 | discovery | 78497 | 69897 | 69897 | 65564 |
| c61 | discovery | 78115 | 68929 | 68929 | 67984 |
| c62 | discovery | 74796 | 69606 | 69606 | 68872 |
| c64 | discovery | 83478 | 75019 | 75019 | 72526 |
| c65 | discovery | 89802 | 79632 | 79632 | 77480 |
| c66 | discovery | 89173 | 79634 | 79634 | 78651 |
| c67 | discovery | 68741 | 60743 | 60743 | 60466 |
| c68 | discovery | 70027 | 61710 | 61710 | 60866 |
| c69 | discovery | 51114 | 45069 | 45069 | 44285 |
| c7 | discovery | 75140 | 67346 | 67346 | 65994 |
| c70 | discovery | 89941 | 83425 | 83425 | 81617 |
| c71 | discovery | 90403 | 80451 | 80451 | 79638 |
| c74 | discovery | 71066 | 53846 | 53846 | 53419 |
| c75 | discovery | 62078 | 49486 | 49486 | 48268 |
| c76 | discovery | 83945 | 77751 | 77751 | 76620 |
| c8 | discovery | 89061 | 77306 | 77306 | 74466 |
| c80 | discovery | 72803 | 59407 | 59407 | 59154 |
| c81 | discovery | 71905 | 57776 | 57776 | 57479 |
| c82 | discovery | 71661 | 64108 | 64108 | 62998 |
| c83 | discovery | 67265 | 53997 | 53997 | 53549 |
| c84 | discovery | 92888 | 72724 | 72724 | 71646 |
| c85 | discovery | 76064 | 60277 | 60277 | 59334 |
| c86 | discovery | 74121 | 64465 | 64465 | 63782 |
| c87 | discovery | 76535 | 60844 | 60844 | 59819 |
| c89 | discovery | 78501 | 62485 | 62485 | 62080 |
| c92 | discovery | 60550 | 48485 | 48485 | 48083 |
| c93 | discovery | 75125 | 60737 | 60737 | 59262 |
| c94 | discovery | 48095 | 37832 | 37832 | 37415 |
| c95 | discovery | 79142 | 65100 | 65100 | 63949 |
| c97 | discovery | 89090 | 69475 | 69475 | 68718 |
| c99 | discovery | 58615 | 46704 | 46704 | 46468 |
| G1 | discovery | 78707 | 64589 | 64589 | 63315 |
| G10 | discovery | 73553 | 59876 | 59876 | 55088 |
| G100 | discovery | 53854 | 42432 | 42432 | 40980 |
| G11 | discovery | 78005 | 67788 | 67788 | 65212 |
| G12 | discovery | 48495 | 40454 | 40454 | 39786 |
| G13 | discovery | 76649 | 66182 | 66182 | 64327 |
| G14 | discovery | 77188 | 66243 | 66243 | 64650 |
| G16 | discovery | 84969 | 73118 | 73118 | 71088 |
| G18 | discovery | 80359 | 70223 | 70223 | 67219 |
| G19 | discovery | 75644 | 62502 | 62502 | 59950 |
| G2 | discovery | 81103 | 72486 | 72486 | 67402 |
| G20 | discovery | 90293 | 77966 | 77966 | 77123 |
| G22 | discovery | 85651 | 72647 | 72647 | 68908 |
| G23 | discovery | 76866 | 67520 | 67520 | 66236 |
| G25 | discovery | 51032 | 41835 | 41835 | 39892 |
| G26 | discovery | 78637 | 64102 | 64102 | 60447 |
| G27 | discovery | 65651 | 52629 | 52629 | 42794 |
| G28 | discovery | 87207 | 71273 | 71273 | 67930 |
| G29 | discovery | 77126 | 63344 | 63344 | 56841 |
| G3 | discovery | 83665 | 71930 | 71930 | 66636 |
| G30 | discovery | 73372 | 63999 | 63999 | 59229 |
| G31 | discovery | 86037 | 71131 | 71131 | 63387 |
| G32 | discovery | 46554 | 38455 | 38455 | 36659 |
| G33 | discovery | 91502 | 74994 | 74994 | 74469 |
| G41 | discovery | 76694 | 61572 | 61572 | 61417 |
| G42 | discovery | 88090 | 72193 | 72193 | 71921 |
| G43 | discovery | 91695 | 74980 | 74980 | 73845 |
| G48 | discovery | 87755 | 69211 | 69211 | 67840 |
| G49 | discovery | 91059 | 73817 | 73817 | 72569 |
| G50 | discovery | 89620 | 72215 | 72215 | 71895 |
| G51 | discovery | 81991 | 66464 | 66464 | 66170 |
| G52 | discovery | 78573 | 69442 | 69442 | 68153 |
| G53 | discovery | 82443 | 65905 | 65905 | 65143 |
| G54 | discovery | 82778 | 73471 | 73471 | 69323 |
| G55 | discovery | 78160 | 69131 | 69131 | 67364 |
| G56 | discovery | 75546 | 69945 | 69945 | 68449 |
| G57 | discovery | 79300 | 73864 | 73864 | 72748 |
| G59 | discovery | 89945 | 83654 | 83654 | 81258 |
| G6 | discovery | 87022 | 75397 | 75397 | 71633 |
| G60 | discovery | 87515 | 77504 | 77504 | 74335 |
| G62 | discovery | 80380 | 71266 | 71266 | 68659 |
| G64 | discovery | 72124 | 66314 | 66314 | 65935 |
| G66 | discovery | 81613 | 75952 | 75952 | 74980 |
| G67 | discovery | 86048 | 79569 | 79569 | 78463 |
| G68 | discovery | 89519 | 78864 | 78864 | 78136 |
| G69 | discovery | 76063 | 67292 | 67292 | 66788 |
| G7 | discovery | 88402 | 77070 | 77070 | 74715 |
| G70 | discovery | 80075 | 70247 | 70247 | 69582 |
| G71 | discovery | 85103 | 78683 | 78683 | 77466 |
| G73 | discovery | 78866 | 73620 | 73620 | 71965 |
| G74 | discovery | 79884 | 56067 | 56067 | 55764 |
| G75 | discovery | 81505 | 75592 | 75592 | 74790 |
| G76 | discovery | 79174 | 61759 | 61759 | 61347 |
| G77 | discovery | 73376 | 58253 | 58253 | 57912 |
| G8 | discovery | 65055 | 53692 | 53692 | 51807 |
| G81 | discovery | 60799 | 48508 | 48508 | 47584 |
| G82 | discovery | 87213 | 69068 | 69068 | 68699 |
| G83 | discovery | 75286 | 59134 | 59134 | 58678 |
| G84 | discovery | 78034 | 63273 | 63273 | 62189 |
| G85 | discovery | 86055 | 69492 | 69492 | 67733 |
| G88 | discovery | 72675 | 57102 | 57102 | 55293 |
| G89 | discovery | 76301 | 61193 | 61193 | 60781 |
| G90 | discovery | 59171 | 47291 | 47291 | 46832 |
| G92 | discovery | 61441 | 50218 | 50218 | 49775 |
| G93 | discovery | 84194 | 65859 | 65859 | 64098 |
| G94 | discovery | 74384 | 59141 | 59141 | 57424 |
| G95 | discovery | 58113 | 46262 | 46262 | 43978 |
| G96 | discovery | 57729 | 44968 | 44968 | 44533 |
| G97 | discovery | 77921 | 50993 | 50993 | 50828 |
| G98 | discovery | 50540 | 40127 | 40127 | 40030 |
| c17 | validation | 46099 | 37989 | 37989 | 37151 |
| c26 | validation | 83871 | 72614 | 72614 | 68960 |
| c29 | validation | 71713 | 61893 | 61893 | 59037 |
| c31 | validation | 74249 | 66708 | 66708 | 64560 |
| c38 | validation | 81743 | 66486 | 66486 | 65906 |
| c4 | validation | 88505 | 76845 | 76845 | 72848 |
| c40 | validation | 79409 | 68183 | 68183 | 67139 |
| c41 | validation | 72570 | 64370 | 64370 | 62507 |
| c43 | validation | 76909 | 63836 | 63836 | 63419 |
| c46 | validation | 82325 | 73088 | 73088 | 70364 |
| c47 | validation | 88561 | 72092 | 72092 | 70791 |
| c48 | validation | 90546 | 73042 | 73042 | 72244 |
| c49 | validation | 89846 | 79129 | 79129 | 77791 |
| c5 | validation | 80704 | 70124 | 70124 | 68544 |
| c53 | validation | 91726 | 74493 | 74493 | 73424 |
| c6 | validation | 79549 | 65082 | 65082 | 63428 |
| c63 | validation | 93885 | 87836 | 87836 | 86845 |
| c72 | validation | 71061 | 62967 | 62967 | 62396 |
| c73 | validation | 47103 | 41736 | 41736 | 39762 |
| c77 | validation | 74759 | 58823 | 58823 | 58166 |
| c78 | validation | 58265 | 51145 | 51145 | 50391 |
| c79 | validation | 65452 | 52558 | 52558 | 52201 |
| c88 | validation | 75738 | 60154 | 60154 | 59386 |
| c9 | validation | 72575 | 64627 | 64627 | 62967 |
| c90 | validation | 50670 | 44480 | 44480 | 44142 |
| c91 | validation | 61609 | 48982 | 48982 | 48408 |
| c96 | validation | 60804 | 47359 | 47359 | 47335 |
| c98 | validation | 84138 | 53330 | 53330 | 53316 |
| G15 | validation | 53526 | 44449 | 44449 | 39554 |
| G17 | validation | 85754 | 70798 | 70798 | 69371 |
| G21 | validation | 57859 | 47474 | 47474 | 46913 |
| G24 | validation | 81487 | 67621 | 67621 | 65918 |
| G34 | validation | 81328 | 71584 | 71584 | 71063 |
| G36 | validation | 75971 | 67100 | 67100 | 66459 |
| G37 | validation | 75797 | 62339 | 62339 | 61752 |
| G38 | validation | 80164 | 67385 | 67385 | 67129 |
| G39 | validation | 83283 | 67717 | 67717 | 67245 |
| G4 | validation | 75517 | 65760 | 65760 | 60187 |
| G40 | validation | 81030 | 65844 | 65844 | 65470 |
| G45 | validation | 87383 | 71393 | 71393 | 70766 |
| G46 | validation | 86575 | 71935 | 71935 | 71633 |
| G47 | validation | 87745 | 73540 | 73540 | 72933 |
| G5 | validation | 80506 | 70522 | 70522 | 67694 |
| G58 | validation | 74153 | 69011 | 69011 | 67768 |
| G61 | validation | 69194 | 61606 | 61606 | 59597 |
| G63 | validation | 60648 | 53363 | 53363 | 52983 |
| G65 | validation | 90751 | 83475 | 83475 | 81351 |
| G72 | validation | 72502 | 63937 | 63937 | 63209 |
| G78 | validation | 79457 | 64275 | 64275 | 63848 |
| G79 | validation | 71309 | 57390 | 57390 | 57095 |
| G80 | validation | 64643 | 51709 | 51709 | 51338 |
| G86 | validation | 78018 | 63467 | 63467 | 62042 |
| G87 | validation | 64591 | 52193 | 52193 | 51141 |
| G9 | validation | 69552 | 58164 | 58164 | 56740 |
| G91 | validation | 63568 | 51178 | 51178 | 50874 |
| G99 | validation | 58752 | 46575 | 46575 | 45061 |

| Supplementary Table 2**. Relative abundance of differential genera in the discovery set** | | | | | | | | | | | | |
| --- | --- | --- | --- | --- | --- | --- | --- | --- | --- | --- | --- | --- |
|  | Dialister | Ruminococcaceae UCG 002 | Eubacterium eligens group | Parabacteroides | Lachnospiraceae NK4A136 group | Megasphaera | Ruminococcaceae UCG 005 | Parasutterella | Eisenbergiella | Tyzzerella 4 | Eubacterium xylanophilum group | Ruminococcaceae UCG 003 |
| c1 | 8.03E-03 | 2.39E-02 | 1.04E-02 | 1.65E-02 | 3.64E-03 | 2.78E-04 | 1.21E-02 | 5.61E-03 | 0 | 0 | 2.28E-03 | 5.31E-03 |
| c10 | 3.53E-03 | 1.09E-02 | 6.17E-03 | 1.28E-02 | 9.67E-03 | 4.72E-04 | 3.25E-03 | 8.64E-03 | 0 | 0 | 1.56E-03 | 2.72E-03 |
| c100 | 6.50E-03 | 6.79E-02 | 7.56E-03 | 7.83E-03 | 4.17E-03 | 2.17E-03 | 8.08E-03 | 1.42E-03 | 0 | 0 | 2.19E-03 | 2.44E-03 |
| c11 | 6.64E-03 | 2.20E-02 | 6.78E-03 | 1.13E-02 | 3.78E-03 | 1.22E-03 | 8.08E-03 | 4.17E-03 | 1.67E-04 | 0 | 2.86E-03 | 3.19E-03 |
| c12 | 5.00E-03 | 1.43E-02 | 7.56E-03 | 1.96E-02 | 8.06E-03 | 4.72E-04 | 4.42E-03 | 4.97E-02 | 1.67E-04 | 2.22E-04 | 2.75E-03 | 2.64E-03 |
| c13 | 5.69E-03 | 2.75E-02 | 1.33E-02 | 2.78E-02 | 7.14E-03 | 1.06E-03 | 8.25E-03 | 4.58E-03 | 1.11E-04 | 0 | 3.58E-03 | 7.61E-03 |
| c14 | 7.14E-03 | 2.07E-02 | 4.94E-03 | 2.25E-02 | 4.83E-03 | 1.46E-02 | 3.42E-03 | 1.03E-02 | 0 | 0 | 6.06E-03 | 4.53E-03 |
| c15 | 2.06E-03 | 1.46E-02 | 1.69E-02 | 1.73E-02 | 3.28E-03 | 5.56E-04 | 4.78E-03 | 9.97E-03 | 0 | 0 | 2.25E-03 | 4.81E-03 |
| c16 | 2.75E-03 | 1.41E-02 | 1.19E-02 | 3.06E-02 | 3.11E-03 | 4.17E-04 | 5.78E-03 | 2.70E-02 | 1.19E-03 | 5.56E-05 | 1.19E-03 | 3.33E-03 |
| c18 | 1.36E-03 | 8.44E-03 | 7.44E-03 | 2.49E-02 | 4.36E-03 | 4.72E-04 | 4.39E-03 | 6.67E-04 | 0 | 0 | 7.22E-04 | 1.31E-03 |
| c19 | 1.47E-03 | 1.06E-02 | 3.92E-03 | 1.09E-02 | 3.36E-03 | 1.11E-04 | 4.75E-03 | 5.43E-02 | 1.06E-03 | 4.72E-04 | 1.25E-03 | 4.53E-03 |
| c2 | 2.25E-03 | 1.41E-02 | 6.92E-03 | 1.38E-02 | 2.11E-03 | 1.67E-04 | 8.53E-03 | 2.93E-02 | 2.78E-04 | 2.22E-04 | 1.86E-03 | 5.61E-03 |
| c20 | 3.28E-03 | 2.25E-02 | 7.69E-03 | 9.67E-03 | 7.33E-03 | 8.33E-05 | 5.78E-03 | 4.64E-03 | 1.11E-04 | 0 | 1.39E-03 | 3.58E-03 |
| c21 | 2.33E-03 | 2.37E-02 | 7.03E-03 | 7.42E-03 | 4.33E-03 | 3.06E-04 | 4.94E-03 | 6.39E-03 | 0 | 0 | 1.31E-03 | 3.17E-03 |
| c22 | 5.33E-03 | 2.83E-02 | 1.18E-02 | 8.19E-03 | 2.53E-03 | 3.33E-04 | 8.36E-03 | 6.86E-03 | 0 | 0 | 8.36E-03 | 5.39E-03 |
| c23 | 1.72E-02 | 2.10E-02 | 3.39E-03 | 1.87E-02 | 1.36E-03 | 1.02E-02 | 8.53E-03 | 3.83E-03 | 6.11E-04 | 0 | 1.72E-03 | 2.31E-03 |
| c24 | 3.94E-03 | 5.72E-03 | 1.81E-03 | 2.36E-02 | 1.17E-03 | 1.28E-02 | 1.83E-03 | 5.31E-03 | 0 | 1.06E-03 | 8.06E-04 | 1.56E-03 |
| c25 | 1.82E-02 | 6.12E-02 | 4.69E-03 | 6.08E-02 | 3.97E-03 | 1.03E-03 | 1.44E-02 | 6.03E-03 | 0 | 0 | 9.72E-04 | 4.58E-03 |
| c27 | 1.78E-03 | 8.22E-03 | 3.60E-02 | 9.72E-03 | 1.03E-03 | 2.22E-04 | 2.19E-03 | 1.03E-03 | 0 | 0 | 3.61E-04 | 3.89E-03 |
| c28 | 6.11E-03 | 1.15E-02 | 2.92E-03 | 1.10E-02 | 5.14E-03 | 2.17E-03 | 4.06E-03 | 5.08E-03 | 8.89E-04 | 2.22E-04 | 4.06E-03 | 2.28E-03 |
| c3 | 4.27E-02 | 4.71E-02 | 3.17E-03 | 1.87E-02 | 5.56E-04 | 2.22E-03 | 6.47E-03 | 1.47E-02 | 0 | 0 | 6.94E-04 | 7.36E-03 |
| c30 | 1.92E-03 | 2.43E-02 | 5.00E-03 | 1.48E-02 | 3.33E-03 | 0 | 3.75E-03 | 3.33E-03 | 0 | 2.22E-04 | 3.25E-03 | 5.47E-03 |
| c32 | 1.30E-01 | 1.08E-01 | 2.58E-03 | 1.99E-02 | 2.31E-03 | 7.25E-03 | 9.58E-03 | 6.39E-03 | 1.11E-04 | 0 | 1.31E-03 | 7.06E-03 |
| c33 | 9.44E-03 | 8.42E-03 | 3.78E-03 | 1.27E-02 | 8.17E-03 | 4.17E-04 | 2.08E-03 | 5.33E-02 | 2.22E-04 | 7.78E-04 | 6.39E-04 | 3.58E-03 |
| c34 | 8.83E-03 | 1.05E-02 | 1.68E-02 | 2.35E-02 | 3.08E-03 | 6.11E-04 | 1.58E-03 | 1.98E-02 | 2.78E-04 | 1.69E-03 | 3.64E-03 | 3.69E-03 |
| c36 | 3.00E-03 | 1.25E-02 | 1.16E-02 | 1.27E-02 | 1.64E-02 | 1.11E-04 | 2.75E-03 | 6.72E-03 | 1.11E-04 | 5.56E-05 | 3.11E-03 | 1.75E-03 |
| c37 | 1.02E-01 | 1.76E-02 | 1.51E-02 | 1.54E-02 | 4.94E-03 | 6.11E-04 | 3.83E-03 | 1.18E-01 | 3.33E-04 | 1.47E-03 | 1.50E-03 | 3.06E-03 |
| c39 | 9.47E-03 | 1.12E-02 | 5.89E-03 | 2.73E-02 | 2.42E-03 | 8.33E-04 | 3.00E-03 | 1.60E-02 | 1.39E-03 | 9.17E-04 | 1.14E-03 | 2.06E-03 |
| c42 | 6.59E-02 | 1.09E-02 | 1.56E-02 | 1.66E-02 | 5.44E-03 | 8.33E-04 | 1.83E-03 | 8.83E-03 | 3.89E-04 | 1.56E-03 | 2.11E-03 | 3.17E-03 |
| c45 | 6.28E-03 | 9.69E-03 | 6.33E-03 | 3.91E-02 | 6.03E-03 | 6.11E-04 | 3.44E-03 | 3.71E-02 | 1.25E-03 | 6.39E-04 | 3.08E-03 | 1.28E-03 |
| c50 | 5.64E-03 | 1.05E-02 | 3.22E-03 | 1.68E-02 | 2.11E-02 | 0 | 3.61E-03 | 1.39E-02 | 1.39E-04 | 0 | 2.28E-03 | 4.08E-03 |
| c51 | 1.83E-02 | 2.12E-02 | 5.14E-03 | 3.11E-02 | 4.08E-03 | 6.94E-04 | 2.86E-03 | 2.10E-02 | 1.11E-03 | 9.17E-04 | 3.14E-03 | 3.14E-03 |
| c52 | 4.50E-03 | 9.61E-03 | 3.07E-02 | 5.16E-02 | 7.75E-03 | 0 | 2.61E-03 | 1.88E-02 | 1.39E-04 | 8.61E-04 | 6.39E-03 | 2.64E-03 |
| c54 | 4.22E-03 | 7.00E-03 | 1.01E-02 | 1.03E-02 | 5.75E-03 | 0 | 2.19E-03 | 4.11E-03 | 0 | 0 | 2.25E-03 | 3.25E-03 |
| c55 | 5.17E-03 | 1.43E-02 | 6.64E-02 | 1.01E-02 | 7.19E-03 | 3.06E-04 | 3.86E-03 | 5.94E-03 | 0 | 0 | 3.36E-03 | 4.28E-03 |
| c56 | 2.54E-02 | 1.09E-02 | 1.73E-02 | 8.58E-03 | 6.36E-03 | 7.50E-04 | 1.97E-03 | 4.14E-03 | 0 | 0 | 2.97E-03 | 3.58E-03 |
| c57 | 2.15E-02 | 1.86E-02 | 8.64E-03 | 1.10E-02 | 1.13E-02 | 0 | 4.17E-03 | 4.25E-03 | 5.56E-05 | 0 | 6.64E-03 | 5.39E-03 |
| c58 | 5.08E-03 | 3.50E-03 | 2.84E-02 | 1.01E-02 | 9.03E-03 | 0 | 4.17E-04 | 1.01E-02 | 8.33E-05 | 0 | 1.03E-03 | 2.06E-03 |
| c59 | 5.11E-03 | 1.19E-02 | 1.48E-02 | 1.08E-02 | 1.83E-02 | 0 | 1.92E-03 | 1.55E-02 | 0 | 0 | 8.83E-03 | 3.78E-03 |
| c60 | 3.08E-03 | 2.19E-02 | 4.56E-03 | 7.00E-03 | 3.06E-03 | 0 | 1.58E-03 | 1.78E-02 | 8.33E-05 | 5.56E-05 | 2.00E-03 | 4.28E-03 |
| c61 | 5.42E-03 | 8.36E-03 | 1.36E-02 | 1.67E-02 | 8.56E-03 | 0 | 1.33E-03 | 7.25E-03 | 0 | 0 | 5.00E-03 | 1.86E-03 |
| c62 | 2.54E-02 | 9.81E-03 | 2.08E-02 | 9.33E-03 | 4.39E-03 | 2.03E-03 | 9.17E-04 | 2.19E-03 | 0 | 0 | 1.00E-03 | 1.94E-03 |
| c64 | 3.05E-02 | 1.01E-02 | 6.75E-03 | 5.56E-03 | 7.53E-03 | 0 | 2.75E-03 | 6.08E-03 | 0 | 0 | 1.36E-03 | 3.31E-03 |
| c65 | 8.14E-03 | 8.00E-03 | 1.04E-02 | 8.67E-03 | 4.67E-03 | 6.94E-04 | 1.56E-03 | 1.69E-03 | 2.78E-05 | 7.50E-04 | 1.06E-03 | 2.33E-03 |
| c66 | 2.58E-03 | 1.10E-02 | 5.86E-03 | 7.72E-03 | 9.17E-04 | 6.39E-04 | 2.94E-03 | 5.36E-03 | 1.67E-04 | 1.00E-03 | 8.06E-04 | 3.33E-03 |
| c67 | 2.75E-02 | 6.11E-03 | 1.44E-03 | 1.08E-02 | 3.33E-04 | 2.88E-02 | 1.44E-03 | 5.56E-03 | 1.94E-04 | 1.20E-02 | 1.86E-03 | 2.78E-03 |
| c68 | 7.00E-03 | 1.56E-02 | 7.19E-03 | 1.90E-02 | 5.00E-03 | 1.72E-03 | 5.47E-03 | 2.57E-02 | 0 | 8.89E-04 | 2.81E-03 | 5.86E-03 |
| c69 | 4.33E-03 | 1.84E-02 | 9.44E-03 | 1.36E-02 | 4.87E-02 | 1.00E-03 | 6.36E-03 | 1.38E-02 | 0 | 0 | 3.75E-03 | 4.14E-03 |
| c7 | 1.69E-03 | 6.94E-03 | 2.89E-03 | 1.61E-02 | 3.92E-03 | 1.39E-04 | 1.78E-03 | 3.56E-03 | 0 | 0 | 7.33E-03 | 1.86E-03 |
| c70 | 1.00E-03 | 3.14E-03 | 4.36E-03 | 2.42E-02 | 6.11E-04 | 5.83E-04 | 1.28E-03 | 5.08E-02 | 8.33E-05 | 2.22E-04 | 1.19E-03 | 3.42E-03 |
| c71 | 1.20E-02 | 1.36E-02 | 3.83E-03 | 1.98E-02 | 4.83E-03 | 8.33E-04 | 2.03E-03 | 1.03E-02 | 0 | 5.00E-04 | 8.06E-04 | 2.75E-03 |
| c74 | 3.69E-03 | 6.92E-03 | 1.43E-02 | 1.78E-02 | 7.44E-03 | 8.06E-04 | 1.94E-03 | 6.11E-03 | 4.72E-04 | 0 | 2.92E-03 | 3.58E-03 |
| c75 | 3.64E-03 | 8.83E-03 | 5.56E-03 | 6.47E-03 | 2.58E-03 | 0 | 3.11E-03 | 1.14E-03 | 1.39E-03 | 2.50E-04 | 1.83E-03 | 1.67E-03 |
| c76 | 3.58E-03 | 1.88E-02 | 5.81E-03 | 1.15E-02 | 1.59E-02 | 1.83E-02 | 1.11E-03 | 1.14E-02 | 0 | 0 | 4.78E-03 | 2.50E-03 |
| c8 | 2.53E-03 | 4.28E-02 | 5.86E-03 | 1.81E-02 | 2.42E-03 | 2.22E-04 | 5.42E-03 | 4.22E-03 | 0 | 0 | 6.08E-03 | 8.25E-03 |
| c80 | 5.39E-02 | 2.60E-02 | 9.42E-03 | 9.00E-03 | 6.72E-03 | 0 | 5.56E-03 | 1.36E-02 | 2.78E-05 | 9.72E-04 | 2.17E-03 | 3.42E-03 |
| c81 | 2.23E-02 | 1.32E-02 | 7.42E-03 | 1.15E-02 | 4.64E-03 | 3.11E-03 | 3.61E-03 | 2.03E-03 | 0 | 0 | 8.06E-04 | 2.36E-03 |
| c82 | 1.01E-02 | 1.63E-02 | 8.31E-03 | 1.97E-02 | 5.89E-03 | 0 | 4.75E-03 | 1.49E-02 | 0 | 1.39E-04 | 1.28E-03 | 2.75E-03 |
| c83 | 6.36E-03 | 1.39E-02 | 3.52E-02 | 1.19E-02 | 6.19E-03 | 0 | 5.14E-03 | 1.54E-02 | 0 | 2.22E-04 | 2.58E-03 | 4.50E-03 |
| c84 | 2.31E-03 | 4.17E-03 | 1.68E-02 | 2.13E-02 | 1.92E-03 | 0 | 5.00E-04 | 1.55E-02 | 5.56E-05 | 1.64E-03 | 1.61E-03 | 1.33E-03 |
| c85 | 7.50E-02 | 7.69E-03 | 2.44E-02 | 1.04E-02 | 5.72E-03 | 0 | 1.44E-03 | 3.75E-03 | 0 | 0 | 6.39E-04 | 2.33E-03 |
| c86 | 1.08E-02 | 8.21E-02 | 3.33E-03 | 1.66E-02 | 3.61E-03 | 8.61E-04 | 3.33E-02 | 4.06E-03 | 1.39E-04 | 4.17E-04 | 1.72E-03 | 5.14E-03 |
| c87 | 3.11E-03 | 9.67E-03 | 3.14E-02 | 1.01E-02 | 8.33E-04 | 8.06E-04 | 1.94E-03 | 1.69E-03 | 0 | 5.83E-04 | 1.71E-02 | 6.25E-03 |
| c89 | 1.15E-02 | 4.54E-02 | 8.00E-03 | 4.56E-02 | 9.11E-03 | 8.33E-05 | 1.01E-02 | 1.25E-02 | 0 | 3.06E-04 | 1.08E-03 | 5.58E-03 |
| c92 | 4.67E-03 | 6.76E-02 | 8.94E-03 | 2.67E-02 | 3.14E-03 | 1.75E-03 | 7.81E-03 | 1.50E-03 | 1.94E-04 | 0 | 4.64E-03 | 2.33E-03 |
| c93 | 6.78E-03 | 4.18E-02 | 7.17E-03 | 8.81E-03 | 3.08E-03 | 4.10E-02 | 2.23E-02 | 7.78E-04 | 0 | 0 | 2.33E-03 | 2.67E-03 |
| c94 | 2.39E-02 | 1.52E-02 | 7.83E-03 | 9.11E-03 | 4.28E-03 | 2.22E-03 | 5.31E-03 | 5.92E-03 | 0 | 0 | 2.25E-03 | 2.19E-03 |
| c95 | 2.41E-02 | 5.45E-02 | 4.75E-03 | 1.15E-02 | 8.33E-04 | 5.00E-04 | 2.52E-02 | 1.11E-03 | 6.11E-04 | 4.36E-03 | 6.11E-04 | 3.61E-04 |
| c97 | 2.42E-02 | 1.27E-02 | 8.06E-03 | 2.50E-02 | 5.65E-02 | 0 | 3.50E-03 | 2.08E-03 | 0 | 2.22E-04 | 1.61E-03 | 3.28E-03 |
| c99 | 1.09E-02 | 2.14E-02 | 1.31E-02 | 8.14E-03 | 1.21E-02 | 3.94E-03 | 1.05E-02 | 3.61E-03 | 0 | 1.94E-04 | 6.67E-03 | 2.58E-03 |
| G1 | 7.44E-03 | 7.15E-02 | 4.44E-03 | 1.08E-02 | 5.75E-03 | 1.39E-04 | 1.76E-02 | 1.06E-02 | 2.22E-04 | 3.08E-03 | 1.14E-03 | 5.97E-03 |
| G10 | 2.69E-03 | 1.53E-02 | 8.83E-03 | 2.28E-02 | 3.25E-03 | 0 | 5.72E-03 | 1.70E-02 | 5.42E-03 | 3.89E-04 | 2.06E-03 | 3.31E-03 |
| G100 | 8.47E-03 | 3.91E-02 | 5.22E-03 | 1.09E-02 | 6.81E-03 | 0 | 1.32E-02 | 2.11E-03 | 2.36E-03 | 6.67E-04 | 3.06E-04 | 2.81E-03 |
| G11 | 8.06E-04 | 7.47E-03 | 9.47E-03 | 1.22E-02 | 1.09E-02 | 8.33E-05 | 6.58E-03 | 1.06E-03 | 1.94E-04 | 2.78E-04 | 7.78E-04 | 1.11E-03 |
| G12 | 2.11E-03 | 1.50E-02 | 1.88E-02 | 5.75E-03 | 2.94E-03 | 0 | 4.92E-03 | 3.25E-03 | 0 | 2.22E-04 | 3.36E-03 | 3.39E-03 |
| G13 | 2.42E-03 | 2.54E-02 | 8.50E-03 | 7.00E-03 | 1.18E-02 | 0 | 8.11E-03 | 4.67E-03 | 3.89E-04 | 1.94E-04 | 2.97E-03 | 4.42E-03 |
| G14 | 1.67E-03 | 1.52E-02 | 1.73E-02 | 1.14E-02 | 3.03E-03 | 1.39E-04 | 4.44E-03 | 2.08E-03 | 0 | 8.33E-05 | 4.72E-04 | 7.06E-03 |
| G16 | 2.00E-03 | 3.67E-03 | 1.75E-03 | 1.54E-02 | 2.47E-03 | 1.50E-03 | 1.08E-03 | 2.50E-03 | 1.39E-04 | 4.28E-03 | 5.56E-04 | 1.33E-03 |
| G18 | 3.61E-04 | 7.33E-03 | 2.58E-03 | 8.64E-03 | 3.67E-03 | 1.11E-04 | 2.33E-03 | 2.09E-02 | 3.33E-04 | 1.33E-03 | 2.11E-03 | 4.50E-03 |
| G19 | 3.89E-04 | 4.69E-03 | 2.36E-02 | 6.53E-03 | 5.01E-02 | 8.33E-05 | 1.94E-03 | 1.23E-02 | 2.78E-04 | 1.14E-03 | 1.57E-02 | 8.33E-04 |
| G2 | 1.24E-02 | 2.04E-02 | 2.07E-02 | 3.23E-02 | 6.83E-03 | 1.11E-04 | 3.78E-03 | 9.69E-03 | 4.72E-04 | 1.83E-03 | 1.39E-03 | 7.53E-03 |
| G20 | 1.72E-03 | 4.56E-03 | 1.00E-03 | 5.94E-03 | 2.61E-03 | 3.94E-03 | 1.81E-03 | 1.47E-03 | 2.31E-03 | 8.61E-04 | 1.56E-03 | 1.00E-03 |
| G22 | 8.28E-03 | 2.96E-02 | 1.36E-03 | 1.99E-02 | 2.75E-03 | 7.78E-04 | 3.11E-03 | 3.17E-03 | 0 | 7.50E-04 | 1.44E-03 | 5.31E-03 |
| G23 | 3.61E-04 | 6.33E-03 | 3.03E-03 | 1.14E-02 | 1.07E-02 | 1.67E-04 | 4.17E-04 | 2.81E-03 | 1.67E-04 | 1.14E-03 | 6.67E-03 | 1.86E-03 |
| G25 | 1.75E-03 | 2.31E-02 | 2.58E-03 | 1.52E-02 | 1.13E-02 | 1.39E-04 | 6.58E-03 | 3.36E-03 | 0 | 3.64E-02 | 1.19E-03 | 3.58E-03 |
| G26 | 7.58E-03 | 1.34E-02 | 9.36E-03 | 2.51E-02 | 7.69E-03 | 3.06E-04 | 1.94E-03 | 7.17E-03 | 3.42E-03 | 8.56E-03 | 2.08E-03 | 5.86E-03 |
| G27 | 1.33E-03 | 4.11E-03 | 2.44E-03 | 5.11E-03 | 9.56E-03 | 1.39E-04 | 8.89E-04 | 4.78E-02 | 0 | 7.50E-04 | 1.53E-03 | 1.64E-03 |
| G28 | 1.20E-02 | 5.14E-03 | 3.11E-03 | 4.39E-03 | 7.64E-03 | 1.94E-04 | 1.64E-03 | 4.28E-03 | 2.22E-04 | 1.17E-03 | 2.58E-03 | 1.22E-03 |
| G29 | 4.44E-04 | 5.00E-03 | 4.64E-03 | 6.25E-03 | 6.64E-03 | 1.39E-04 | 1.53E-03 | 3.19E-03 | 3.06E-04 | 1.11E-03 | 1.11E-03 | 1.83E-03 |
| G3 | 3.25E-03 | 5.33E-03 | 3.19E-03 | 6.00E-03 | 4.42E-03 | 8.33E-05 | 1.94E-03 | 9.36E-03 | 0 | 8.33E-05 | 6.94E-04 | 1.89E-03 |
| G30 | 1.19E-03 | 9.81E-03 | 3.25E-03 | 7.72E-03 | 4.14E-02 | 1.11E-04 | 7.81E-03 | 4.97E-03 | 4.44E-04 | 1.19E-03 | 6.78E-03 | 1.64E-03 |
| G31 | 2.36E-02 | 7.36E-03 | 3.03E-03 | 7.44E-03 | 8.36E-03 | 3.61E-04 | 2.58E-03 | 3.89E-03 | 2.22E-04 | 1.28E-03 | 1.86E-03 | 2.92E-03 |
| G32 | 1.49E-02 | 9.78E-03 | 2.06E-03 | 1.13E-02 | 5.00E-03 | 1.81E-03 | 1.25E-03 | 3.50E-03 | 3.33E-04 | 9.17E-04 | 4.50E-03 | 2.42E-03 |
| G33 | 3.31E-02 | 3.24E-02 | 3.39E-03 | 1.59E-02 | 6.39E-03 | 2.78E-05 | 6.97E-03 | 1.09E-02 | 1.39E-04 | 1.39E-04 | 3.75E-03 | 2.33E-03 |
| G41 | 4.06E-03 | 6.53E-03 | 1.35E-02 | 1.60E-02 | 7.44E-03 | 0 | 1.53E-03 | 9.25E-03 | 2.50E-04 | 2.78E-04 | 4.58E-03 | 2.22E-03 |
| G42 | 7.33E-03 | 8.42E-03 | 2.78E-03 | 8.81E-03 | 1.18E-02 | 0 | 2.58E-03 | 1.20E-02 | 1.11E-04 | 0 | 1.75E-03 | 2.72E-03 |
| G43 | 3.44E-03 | 9.69E-03 | 3.36E-02 | 1.12E-02 | 1.24E-02 | 0 | 1.78E-03 | 9.03E-03 | 2.50E-04 | 0 | 2.72E-03 | 2.44E-03 |
| G48 | 5.36E-03 | 1.26E-02 | 1.09E-02 | 1.79E-02 | 4.81E-03 | 2.22E-04 | 3.00E-03 | 1.09E-02 | 5.00E-04 | 3.33E-04 | 1.58E-03 | 3.25E-03 |
| G49 | 8.33E-04 | 5.39E-03 | 5.75E-03 | 2.28E-02 | 2.86E-03 | 8.33E-05 | 8.33E-04 | 2.69E-03 | 7.78E-04 | 2.22E-04 | 6.94E-04 | 1.69E-03 |
| G50 | 1.29E-02 | 5.92E-03 | 7.06E-03 | 3.03E-02 | 4.53E-03 | 2.22E-04 | 1.47E-03 | 2.39E-03 | 7.50E-04 | 7.22E-04 | 1.31E-03 | 2.19E-03 |
| G51 | 5.81E-03 | 5.86E-03 | 2.97E-03 | 2.90E-02 | 4.72E-04 | 1.39E-03 | 1.78E-03 | 4.64E-03 | 2.83E-03 | 2.78E-04 | 5.28E-04 | 8.89E-04 |
| G52 | 4.00E-03 | 7.44E-03 | 8.89E-04 | 1.88E-02 | 2.25E-03 | 5.56E-05 | 1.22E-03 | 9.61E-03 | 9.72E-04 | 2.78E-05 | 1.94E-03 | 1.19E-03 |
| G53 | 1.40E-02 | 2.56E-03 | 9.19E-03 | 2.44E-02 | 1.11E-03 | 4.17E-04 | 8.06E-04 | 6.11E-03 | 8.33E-04 | 3.61E-04 | 1.86E-03 | 1.31E-03 |
| G54 | 2.67E-03 | 7.94E-03 | 5.17E-03 | 1.53E-02 | 1.46E-02 | 0 | 8.33E-04 | 1.08E-01 | 3.06E-04 | 0 | 1.03E-03 | 2.19E-03 |
| G55 | 5.03E-02 | 2.89E-03 | 3.89E-03 | 1.01E-02 | 2.08E-03 | 2.28E-03 | 5.83E-04 | 8.29E-02 | 2.22E-04 | 1.33E-03 | 9.17E-04 | 2.92E-03 |
| G56 | 1.78E-03 | 3.86E-03 | 1.73E-02 | 1.29E-02 | 7.00E-03 | 8.33E-05 | 1.36E-03 | 4.42E-03 | 8.33E-05 | 8.33E-05 | 1.89E-03 | 4.39E-03 |
| G57 | 4.25E-03 | 9.64E-03 | 9.25E-03 | 2.14E-02 | 1.02E-02 | 5.56E-05 | 7.22E-04 | 3.89E-02 | 0 | 0 | 1.31E-03 | 2.25E-03 |
| G59 | 1.58E-03 | 3.47E-03 | 1.15E-02 | 9.78E-03 | 6.92E-03 | 0 | 2.78E-04 | 5.17E-03 | 0 | 0 | 7.44E-03 | 2.58E-03 |
| G6 | 2.97E-03 | 1.33E-02 | 6.97E-03 | 1.21E-02 | 1.27E-02 | 1.11E-04 | 4.03E-03 | 1.11E-02 | 3.33E-04 | 3.06E-04 | 3.75E-03 | 3.69E-03 |
| G60 | 4.83E-03 | 6.83E-03 | 2.44E-02 | 4.83E-03 | 2.36E-02 | 1.67E-04 | 2.83E-03 | 4.61E-03 | 0 | 1.11E-04 | 9.17E-04 | 1.92E-03 |
| G62 | 2.47E-03 | 7.75E-03 | 1.96E-02 | 1.48E-02 | 3.44E-02 | 0 | 2.06E-03 | 8.97E-03 | 1.11E-04 | 1.39E-04 | 8.61E-04 | 3.97E-03 |
| G64 | 1.83E-03 | 3.36E-03 | 4.67E-03 | 6.67E-03 | 2.47E-03 | 0 | 5.83E-04 | 3.58E-03 | 0 | 6.67E-04 | 1.06E-03 | 1.72E-03 |
| G66 | 4.86E-03 | 1.03E-02 | 5.92E-03 | 1.14E-02 | 3.14E-03 | 3.33E-03 | 2.31E-03 | 5.36E-03 | 0 | 6.39E-04 | 3.31E-03 | 3.64E-03 |
| G67 | 4.67E-03 | 8.31E-03 | 6.56E-03 | 7.67E-03 | 3.36E-03 | 9.72E-04 | 2.81E-03 | 9.03E-03 | 0 | 2.78E-04 | 2.69E-03 | 3.17E-03 |
| G68 | 1.50E-03 | 5.56E-03 | 7.25E-03 | 2.05E-02 | 7.06E-03 | 6.11E-04 | 2.42E-03 | 8.31E-03 | 0 | 3.06E-04 | 1.89E-03 | 5.17E-03 |
| G69 | 3.17E-02 | 5.86E-03 | 4.61E-03 | 1.61E-02 | 2.03E-03 | 7.50E-04 | 1.69E-03 | 3.04E-02 | 8.33E-05 | 5.56E-04 | 8.33E-04 | 2.03E-03 |
| G7 | 3.58E-03 | 2.01E-02 | 5.39E-03 | 1.57E-02 | 1.21E-02 | 1.67E-04 | 5.58E-03 | 3.42E-03 | 1.94E-04 | 2.22E-04 | 1.61E-03 | 8.97E-03 |
| G70 | 8.94E-03 | 1.46E-02 | 7.69E-03 | 1.35E-02 | 6.47E-03 | 4.67E-03 | 2.00E-03 | 5.47E-03 | 5.56E-05 | 5.28E-04 | 2.06E-03 | 2.86E-03 |
| G71 | 2.67E-03 | 1.45E-02 | 1.32E-02 | 1.31E-02 | 1.21E-02 | 1.03E-03 | 4.19E-03 | 1.17E-02 | 3.06E-04 | 0 | 9.17E-04 | 4.39E-03 |
| G73 | 2.36E-03 | 1.22E-02 | 5.19E-03 | 6.92E-03 | 6.81E-03 | 3.61E-04 | 2.67E-03 | 4.03E-03 | 0 | 0 | 1.17E-03 | 3.36E-03 |
| G74 | 1.81E-03 | 3.28E-03 | 2.36E-03 | 1.00E-02 | 1.75E-03 | 1.44E-03 | 6.94E-04 | 2.22E-03 | 1.19E-03 | 4.44E-04 | 1.14E-03 | 1.92E-03 |
| G75 | 1.25E-03 | 5.94E-03 | 5.94E-03 | 2.69E-02 | 1.57E-02 | 5.56E-04 | 8.06E-04 | 5.14E-02 | 0 | 0 | 6.67E-04 | 1.11E-03 |
| G76 | 4.42E-03 | 6.33E-03 | 7.89E-03 | 7.11E-03 | 4.06E-03 | 0 | 1.42E-03 | 2.28E-03 | 0 | 5.00E-04 | 2.69E-03 | 2.14E-03 |
| G77 | 1.51E-02 | 1.31E-02 | 5.86E-03 | 1.30E-02 | 7.64E-03 | 3.06E-04 | 5.50E-03 | 2.78E-03 | 3.31E-03 | 3.44E-03 | 1.44E-03 | 2.78E-03 |
| G8 | 5.75E-03 | 2.61E-02 | 3.89E-03 | 1.68E-02 | 6.17E-03 | 2.03E-03 | 4.33E-03 | 8.14E-03 | 0 | 1.67E-04 | 6.50E-03 | 5.08E-03 |
| G81 | 7.00E-03 | 3.09E-02 | 1.11E-02 | 1.14E-02 | 7.25E-03 | 0 | 7.94E-03 | 2.97E-03 | 0 | 8.61E-04 | 2.17E-03 | 3.06E-03 |
| G82 | 9.44E-03 | 4.05E-02 | 1.56E-02 | 1.94E-02 | 1.90E-02 | 0 | 3.19E-02 | 4.44E-03 | 1.39E-04 | 2.50E-04 | 1.56E-03 | 5.50E-03 |
| G83 | 6.00E-03 | 9.25E-03 | 7.25E-03 | 1.37E-02 | 2.21E-02 | 1.94E-04 | 3.06E-03 | 2.00E-03 | 1.86E-03 | 2.50E-04 | 1.14E-03 | 2.14E-03 |
| G84 | 1.07E-02 | 9.78E-03 | 1.38E-02 | 1.06E-02 | 2.11E-02 | 0 | 1.92E-03 | 1.94E-03 | 0 | 4.17E-04 | 6.39E-04 | 1.97E-03 |
| G85 | 3.50E-03 | 1.89E-02 | 8.44E-03 | 1.48E-02 | 5.25E-03 | 4.17E-04 | 2.14E-03 | 1.69E-03 | 0 | 1.39E-04 | 6.39E-04 | 2.19E-03 |
| G88 | 6.89E-03 | 1.06E-02 | 1.57E-02 | 1.70E-02 | 3.60E-02 | 1.11E-04 | 1.58E-03 | 1.22E-03 | 0 | 4.17E-04 | 4.72E-04 | 3.11E-03 |
| G89 | 1.02E-02 | 2.64E-02 | 4.47E-03 | 1.07E-02 | 4.53E-03 | 2.53E-03 | 1.38E-02 | 5.11E-03 | 3.03E-03 | 5.56E-04 | 1.58E-03 | 6.42E-03 |
| G90 | 6.50E-03 | 1.44E-02 | 2.00E-02 | 7.50E-03 | 5.17E-03 | 1.42E-03 | 4.22E-03 | 2.69E-03 | 0 | 0 | 5.19E-03 | 2.83E-03 |
| G92 | 1.48E-02 | 1.39E-02 | 5.06E-03 | 6.78E-03 | 4.56E-03 | 1.03E-03 | 3.94E-03 | 2.64E-03 | 0 | 1.11E-04 | 1.78E-03 | 1.69E-03 |
| G93 | 7.36E-03 | 2.28E-02 | 4.36E-03 | 1.49E-02 | 9.86E-03 | 8.89E-04 | 3.97E-03 | 6.94E-04 | 1.39E-04 | 0 | 1.03E-03 | 2.81E-03 |
| G94 | 6.56E-03 | 1.04E-02 | 3.06E-03 | 7.50E-03 | 8.78E-03 | 0 | 3.47E-03 | 6.11E-04 | 8.89E-04 | 2.78E-04 | 6.67E-04 | 1.50E-03 |
| G95 | 5.25E-03 | 2.03E-02 | 3.86E-03 | 1.00E-01 | 3.89E-03 | 2.50E-04 | 7.47E-03 | 1.58E-03 | 3.86E-02 | 8.06E-04 | 7.22E-04 | 1.78E-03 |
| G96 | 9.25E-03 | 1.25E-02 | 1.36E-03 | 1.19E-02 | 2.83E-03 | 0 | 2.61E-03 | 1.64E-03 | 1.06E-03 | 2.78E-03 | 5.00E-04 | 1.83E-03 |
| G97 | 4.50E-03 | 6.67E-03 | 1.78E-03 | 7.89E-03 | 1.06E-03 | 0 | 3.78E-03 | 1.04E-01 | 6.39E-04 | 1.69E-03 | 3.33E-04 | 3.61E-04 |
| G98 | 1.08E-02 | 2.35E-02 | 2.05E-02 | 4.92E-03 | 2.64E-03 | 3.53E-03 | 6.78E-03 | 1.33E-03 | 2.47E-03 | 5.83E-04 | 2.69E-03 | 2.19E-03 |

| Supplementary Table 3. **Statistical results of differential genera in the discovery set** | | | | | |
| --- | --- | --- | --- | --- | --- |
|  | p value* | LDA score | median_control | median_GDM | score revised |
| Dialister | 0.013 | 3.17 | 6.19E-03 | 4.58E-03 | -3.17 |
| Ruminococcaceae UCG 002 | 0.001 | 3.16 | 1.40E-02 | 9.74E-03 | -3.16 |
| Eubacterium eligens group | 0.029 | 2.73 | 7.56E-03 | 5.81E-03 | -2.73 |
| Parabacteroides | 0.039 | 2.68 | 1.37E-02 | 1.17E-02 | -2.68 |
| Lachnospiraceae NK4A136 group | 0.023 | 2.65 | 4.51E-03 | 6.72E-03 | 2.65 |
| Megasphaera | 0.027 | 2.56 | 5.28E-04 | 1.39E-04 | -2.56 |
| Ruminococcaceae UCG 005 | 0.010 | 2.43 | 3.68E-03 | 2.50E-03 | -2.43 |
| Parasutterella | 0.040 | 2.41 | 6.25E-03 | 4.53E-03 | -2.41 |
| Eisenbergiella | 0.002 | 2.26 | 0 | 2.08E-04 | 2.26 |
| Tyzzerella 4 | 0.000 | 2.16 | 0 | 3.75E-04 | 2.16 |
| Eubacterium xylanophilum group | 0.018 | 2.10 | 2.14E-03 | 1.54E-03 | -2.10 |
| Ruminococcaceae UCG 003 | 0.006 | 2.06 | 3.26E-03 | 2.43E-03 | -2.06 |
| *, *P* values of Kruskal-Wallis test. LDA, linear discriminant analysis. | | | | | |
|  |  |  |  |  |  |

| Supplementary Table 4. **Correlations between maternal clinical indices and differential genera*** | | | | | | | | | | | | | | | | | | | | | | | |
| --- | --- | --- | --- | --- | --- | --- | --- | --- | --- | --- | --- | --- | --- | --- | --- | --- | --- | --- | --- | --- | --- | --- | --- |
|  | BMI | SBP | DBP | Glu | Ins | HGB | TG | TCHOL | HDLCH | LDLCH | ALB | ALT | AST | GGT | CREA | UA | UREA | FT4 | TSH | TPOAB | Leptin | Adiponectin | hsCRP |
| Dialister | 0 | 0 | 0 | 0 | -0.232 | 0 | 0 | 0 | 0 | 0 | 0 | -0.212 | 0 | -0.217 | 0 | -0.172 | 0 | 0 | 0 | 0 | 0 | 0 | 0 |
| Ruminococcaceae UCG 002 | -0.203 | 0 | 0 | -0.171 | 0 | 0 | 0 | 0 | 0 | 0 | 0 | 0 | 0 | 0 | 0 | -0.188 | 0 | 0 | 0 | 0 | 0 | 0 | 0 |
| Eubacterium eligens group | 0 | -0.291 | -0.174 | 0 | 0 | 0 | 0 | 0 | 0 | 0 | 0 | 0 | 0 | 0 | 0 | 0 | 0 | 0 | 0 | 0.169 | 0 | 0 | 0 |
| Parabacteroides | 0 | 0 | 0 | -0.200 | 0 | 0 | 0 | 0 | 0 | 0 | 0 | 0 | 0 | 0 | 0 | 0 | 0.232 | 0 | 0 | 0 | 0 | 0 | 0 |
| Lachnospiraceae NK4A136 group | 0 | 0 | 0 | 0 | 0 | 0 | 0 | 0 | 0 | 0 | 0 | 0 | 0 | 0 | 0 | 0 | 0 | 0 | 0 | 0 | 0 | 0 | 0 |
| Megasphaera | 0 | 0 | 0 | 0 | 0 | 0 | 0 | 0 | 0 | 0 | 0 | 0 | -0.172 | 0 | 0 | 0 | 0 | 0 | 0 | 0 | 0 | 0 | 0 |
| Ruminococcaceae UCG 005 | 0 | 0 | 0 | 0 | 0 | 0 | -0.171 | 0 | 0.167 | 0 | 0 | 0 | 0 | 0 | 0 | -0.209 | 0 | 0 | 0 | 0 | 0 | 0 | 0 |
| Parasutterella | 0 | 0 | 0 | -0.177 | 0 | 0 | 0 | 0 | -0.186 | 0 | 0 | 0 | 0 | 0.168 | 0 | 0 | 0 | 0 | 0 | 0 | -0.206 | 0 | 0 |
| Eisenbergiella | 0 | 0 | 0 | 0.185 | 0 | 0 | 0 | 0 | 0 | 0 | 0 | 0 | 0 | 0 | 0 | 0 | 0 | 0 | 0 | 0 | 0 | 0 | 0 |
| Tyzzerella 4 | 0 | 0 | 0 | 0.194 | 0 | 0 | 0 | 0 | 0 | 0 | 0 | 0 | 0 | 0 | 0 | 0 | 0.188 | 0 | 0 | 0 | 0 | 0 | 0 |
| Eubacterium xylanophilum group | 0 | -0.269 | 0 | 0 | 0 | 0 | 0 | 0 | 0 | 0 | 0 | 0 | 0 | 0 | 0 | 0 | 0 | 0 | 0 | 0 | -0.276 | NA | 0 |
| Ruminococcaceae UCG 003 | -0.178 | 0 | 0 | 0 | 0 | 0 | 0 | 0.204 | 0 | 0 | 0 | 0 | 0 | 0 | 0 | -0.200 | 0 | 0 | 0 | 0 | 0 | 0 | 0 |

*, The r values with corresponding *P* values (Spearman's rank correlation) greater than 0.05 are meaningless, and are set to zero.

Abbreviations: ALB, albumin; ALT, alanine aminotransferase; AST, aspartate aminotransferase; BMI, body mass index; CREA: Creatinine; DBP, diastolic pressure; Glu, fasting blood glucose; FT4: free thyroxine; GGT, glutamine transpeptidase; HDLCH, high density cholesterol; HGB, hemoglobin; hsCRP, high-sensitivity C-reactive protein; Ins, fasting insulin; LDLCH, low density cholesterol; NA, not applied; SBP, systolic pressure; TCHOL, total cholesterol; TG, triglycerides; TPOAB: thyroid peroxidase antibody; TSH: thyroid stimulating hormone; UA, uric acid; UREA: urea.

| Supplementary Table 5**. Correlations between maternal dietary intake and differential genera*** | | | | | | | | | | | | | | | |
| --- | --- | --- | --- | --- | --- | --- | --- | --- | --- | --- | --- | --- | --- | --- | --- |
|  | Daily cereal intake | Daily tuber intake | Daily vegetable intake | Daily fruit intake | Daily meat intake | Daily seafood intake | Daily fresh water products intake | Daily eggs intake | Daily milk intake | Daily beans intake | Daily nuts intake | Daily oil intake | Daily salt intake | Daily water intake | Daily yoghurt intake |
| Dialister | 0 | 0 | 0 | 0 | 0 | 0 | 0 | 0 | 0 | 0 | 0 | -0.184 | 0 | 0 | -0.189 |
| Ruminococcaceae UCG 002 | 0 | 0 | 0 | 0 | 0 | 0 | 0 | 0 | 0 | 0 | 0 | 0 | 0 | 0 | 0 |
| Eubacterium eligens group | 0 | 0 | 0 | 0 | 0 | 0 | 0 | 0 | 0 | 0 | 0 | 0 | 0 | 0 | 0 |
| Parabacteroides | 0 | 0 | 0 | 0 | 0 | 0 | 0 | 0 | 0 | 0 | 0 | -0.248 | 0 | 0 | 0.185 |
| Lachnospiraceae NK4A136 group | 0 | 0 | 0 | 0 | 0 | 0 | 0 | 0 | 0 | 0 | 0 | 0 | 0 | 0 | 0 |
| Megasphaera | 0 | 0 | 0 | 0 | 0 | 0 | 0 | 0 | 0 | 0 | 0 | 0 | 0 | 0 | 0 |
| Ruminococcaceae UCG 005 | 0 | 0 | 0 | 0 | 0 | 0 | 0 | 0 | 0 | 0 | 0 | 0 | 0 | -0.175 | 0 |
| Parasutterella | 0 | 0.187 | 0 | 0 | 0 | 0 | 0 | 0 | 0 | 0 | 0 | 0 | 0 | 0 | 0 |
| Eisenbergiella | 0 | 0 | 0 | 0 | 0 | 0 | 0 | 0 | 0 | 0 | 0 | 0 | 0 | 0 | 0 |
| Tyzzerella 4 | 0 | 0 | 0 | 0 | 0 | 0 | 0 | 0 | 0 | 0 | 0 | 0 | 0 | 0 | 0 |
| Eubacterium xylanophilum group | 0 | 0 | 0 | 0 | 0 | 0 | 0 | 0 | 0 | 0 | 0 | 0 | 0 | 0 | 0 |
| Ruminococcaceae UCG 003 | 0 | 0 | 0 | 0 | 0 | 0 | 0 | 0 | 0 | 0 | 0 | 0 | 0 | 0 | 0.219 |
| *, The r values with corresponding P values (Spearman's rank correlation) greater than 0.05 are meaningless, and are set to zero. | | | | | | | | | | | | | | | |

| Supplementary Table 6**. Correlations between differential genera and the inferred metabolic pathways*** | | | | | | | |
| --- | --- | --- | --- | --- | --- | --- | --- |
|  | Pentose and glucuronate interconversions (ko00040) | Polyketide sugar unit biosynthesis (ko00523) | Starch and sucrose metabolism (ko00500) | Sphingolipid metabolism (ko00600) | Nitrogen metabolism (ko00910) | Lysine biosynthesis (ko00300) |  |
| Dialister | -0.253 | -0.184 | -0.217 | -0.256 | 0.447 | 0.174 |  |
| Lachnospiraceae NK4A136 group | 0 | 0 | 0 | 0 | -0.220 | 0 |  |
| Ruminococcaceae UCG 005 | 0 | 0 | 0 | -0.200 | 0 | 0 |  |
| Eubacterium eligens group | 0 | 0 | 0 | 0 | 0 | 0 |  |
| Eubacterium xylanophilum group | 0 | 0 | 0 | 0 | 0 | 0 |  |
| Eisenbergiella | 0 | 0 | 0 | 0 | 0 | -0.180 |  |
| Tyzzerella 4 | 0 | 0 | 0 | 0 | 0 | -0.187 |  |
| Ruminococcaceae UCG 002 | 0 | 0 | 0 | 0 | 0.204 | 0.312 |  |
| Ruminococcaceae UCG 003 | 0 | 0 | 0 | 0 | 0.249 | 0.270 |  |
| Megasphaera | 0 | 0 | -0.262 | -0.171 | 0.197 | 0.182 |  |
| Parabacteroides | 0 | 0 | 0 | 0 | 0 | 0 |  |
| Parasutterella | 0 | 0 | 0 | 0 | 0 | 0.220 |  |

*, The r values with corresponding *P* values (Spearman's rank correlation) greater than 0.05 are meaningless, and are set to zero.

| Supplementary Table 7**. Performance of models based on bacteria markers, clinical indices and combined markers** | | | | | | | |
| --- | --- | --- | --- | --- | --- | --- | --- |
|  | Discovery set | | |  | Validation set | | |
|  | OTUs^*^ | Clinical indices^†^ | Combined |  | OTUs^*^ | Clincal indices^†^ | Combined |
| Accuracy | 0.707 | 0.700 | 0.736 |  | 0.661 | 0.678 | 0.696 |
| Sensitivity | 0.786 | 0.643 | 0.657 |  | 0.571 | 0.678 | 0.643 |
| Specificity | 0.629 | 0.757 | 0.800 |  | 0.750 | 0.678 | 0.750 |
| AUC | 0.707 | 0.700 | 0.736 |  | 0.661 | 0.678 | 0.696 |

^*^, OTUs were genera *Alloprevotella*, *Ruminococcus 2*, *Parabacteroides*, *Ruminococcaceae UCG-014* and *Uncultured-Ruminococcaceae*.

^†^, Clincal indices were fasting blood glucose and glutamine transpeptidase.

AUC, the areas under the receiver operating characteristic curves.

| Supplementary Table 8**. Performance of the clinical risk-prediction models to identify women who developed GDM** | | | |  |  |
| --- | --- | --- | --- | --- | --- |
| **Model** | **Methodology** | **Clinical risk factors** | **AUC GDM (95% CI)** | | **Original cohort AUC GDM (95% CI)*** |
|  |  |  | Discovery set | Validation set |  |
| Naylor et al. [50] | Cohort of 3131 women (113 GDM) Derivation: 1560 women (44 GDM) Validation: 1571 women (69 GDM) | Maternal age, BMI before pregnancy, ethnicity | 0.539 (0.443-0.635) | 0.787 (0.666-0.908) | 0.729 (0.672–0.785) |
| Van Leeuwen et al. [51] | Cohort of 995 women (24 GDM) | BMI before pregnancy, ethnicity, family history of diabetes, previous GDM | 0.572 (0.475-0.668) | 0.811 (0.698-0.923) | 0.770 (0.690–0.850) |
| Teede et al. [52] | Cohort of 4276 women (356 GDM) Derivation: 2880 women (250 GDM) Validation: 1396 women (106 GDM) | Maternal age, BMI at first visit, ethnicity, family history of diabetes, previous GDM | 0.552 (0.457-0.647) | 0.752 (0.625-0.879) | 0.703 (0.646–0.759) |
| Gabbay-Benziv et al. [53] | Cohort of 924 women (63 GDM) | Maternal age, prior GDM, SBP, first trimester BMI, race | 0.631 (0.537-0.724) | 0.783 (0.664-0.903) | 0.819 (0.769-0.868) |
| *, AUC and CI calculated from data in the original articles.  Abbreviations: AUC, the areas under the receiver operating characteristic curves; BMI, body mass index; CI, confidence interval; GDM, gestational diabetes mellitus; SBP, systolic pressure. | | | | | |
|  |  |  |  |  |  |
|  |  |  |  |  |  |
|  |  |  |  |  |  |

# Supplementary Fig. 1

**
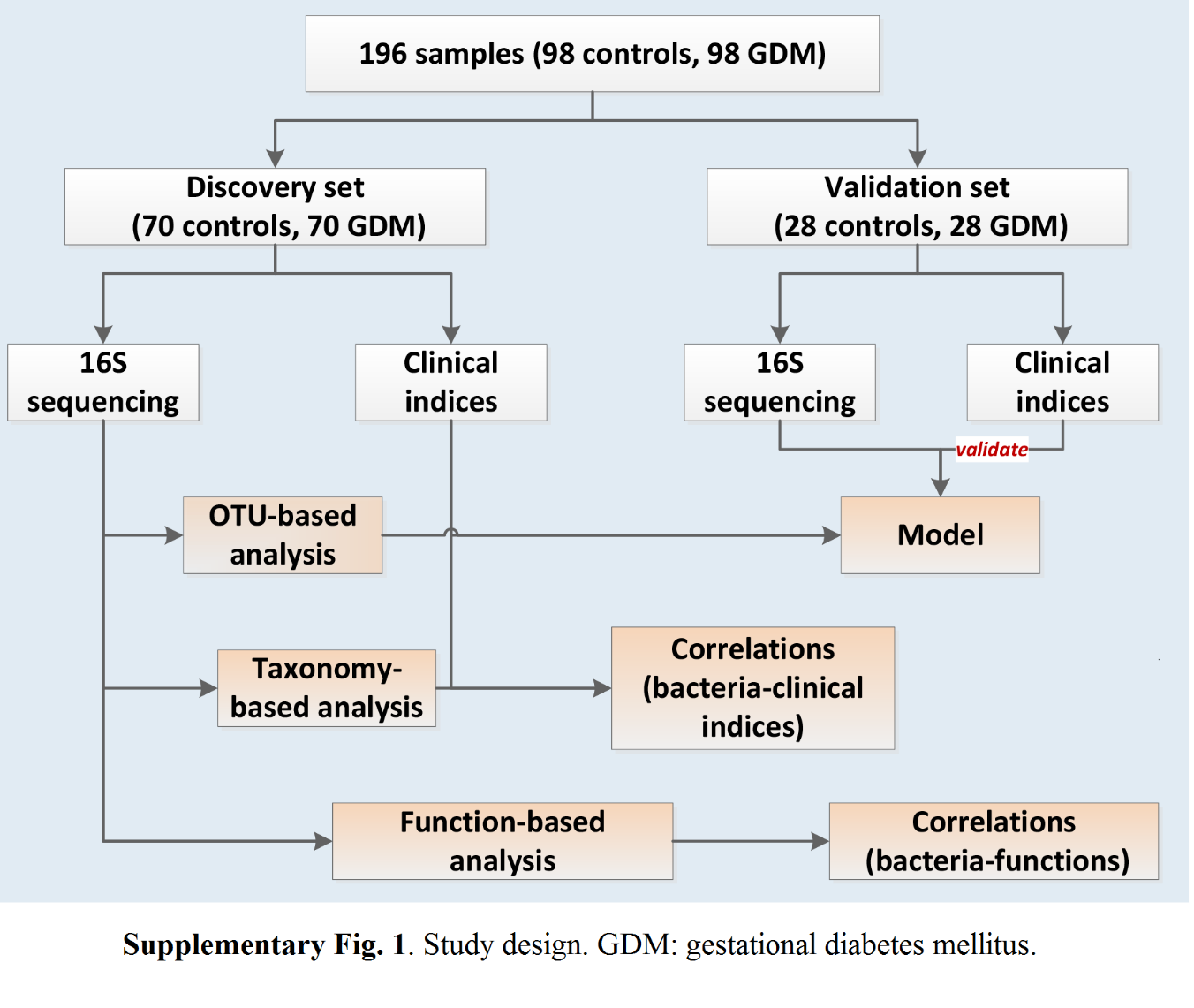
**

# Supplementary Fig. 2

**
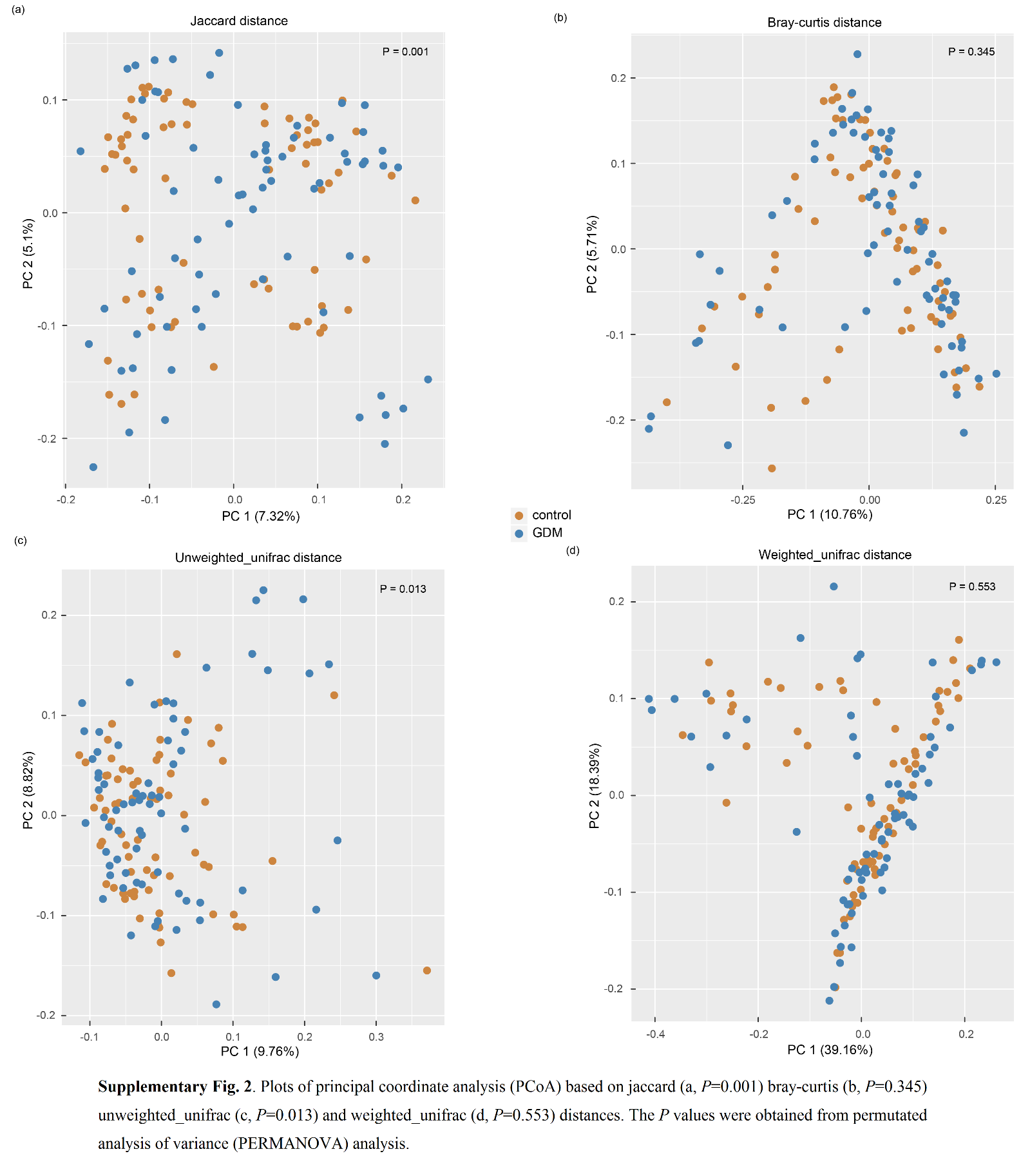
**
